# Supplementary material for: Sexual and reproductive health service utilization of young girls in rural Ethiopia: What are the roles of health extension workers? Community-based cross-sectional study
Source: BMJ Open. 2022 Sep 21;12(9):e056639. doi: 10.1136/bmjopen-2021-056639 (PMC9494555; doi:10.1136/bmjopen-2021-056639)
Supplement: Author's manuscript [file bmjopen-2021-056639.draft_revisions.pdf]

BMJ Open is committed to open peer review. As part of this commitment we make the peer review history of every article we publish publicly available.

When an article is published we post the peer reviewers' comments and the authors' responses online. We also post the versions of the paper that were used during peer review. These are the versions that the peer review comments apply to.

The versions of the paper that follow are the versions that were submitted during the peer review process. They are not the versions of record or the final published versions. They should not be cited or distributed as the published version of this manuscript.

BMJ Open is an open access journal and the full, final, typeset and author-corrected version of record of the manuscript is available on our site with no access controls, subscription charges or pay-per-view fees (http://bmjopen.bmj.com).

If you have any questions on BMJ Open's open peer review process please email info.bmjopen@bmj.com

BMJ Open

Sexual and reproductive health service utilization of girls and young women in rural Ethiopia: What are the roles of health extension workers?: Community based cross sectional study

Journal:	*BMJ Open*
Manuscript ID	bmjopen-2021-056639
Article Type:	Original research
Date Submitted by the Author:	28-Aug-2021
Complete List of Authors:	Jisso, Meskerem ; Hawassa University, Public health<br>Feyasa, Merga; Addis Ababa University College of Natural Sciences, Statistics<br>Medhin, Girmay; Addis Ababa University, Aklilu Lemma Institute of Pathology<br>Dadi, Tegene ; Hawassa University College of Medicine and Health Sciences, School of Public health<br>Simachew, Yilkal; Hawassa University College of Medicine and Health Sciences, School of public health<br>Denberu, Bisrat; federal ministry of health<br>Jebena, Mulusew; Jimma University College of Public Health and Medical Sciences<br>Alemayehun, Yibeltal ; MERQ Consultancy PLC<br>Teklu, Alula M.; MERQ Consultancy PLC ; MERQ PLC LLC, research
Keywords:	REPRODUCTIVE MEDICINE, PUBLIC HEALTH, SEXUAL MEDICINE

SCHOLARONE™
Manuscripts

I, the Submitting Author has the right to grant and does grant on behalf of all authors of the Work (as defined in the below author licence), an exclusive licence and/or a non-exclusive licence for contributions from authors who are: i) UK Crown employees; ii) where BMJ has agreed a CC-BY licence shall apply, and/or iii) in accordance with the terms applicable for US Federal Government officers or employees acting as part of their official duties; on a worldwide, perpetual, irrevocable, royalty-free basis to BMJ Publishing Group Ltd ("BMJ") its licensees and where the relevant Journal is co-owned by BMJ to the co-owners of the Journal, to publish the Work in this journal and any other BMJ products and to exploit all rights, as set out in our licence.

The Submitting Author accepts and understands that any supply made under these terms is made by BMJ to the Submitting Author unless you are acting as an employee on behalf of your employer or a postgraduate student of an affiliated institution which is paying any applicable article publishing charge ("APC") for Open Access articles. Where the Submitting Author wishes to make the Work available on an Open Access basis (and intends to pay the relevant APC), the terms of reuse of such Open Access shall be governed by a Creative Commons licence – details of these licences and which Creative Commons licence will apply to this Work are set out in our licence referred to above.

Other than as permitted in any relevant BMJ Author's Self Archiving Policies, I confirm this Work has not been accepted for publication elsewhere, is not being considered for publication elsewhere and does not duplicate material already published. I confirm all authors consent to publication of this Work and authorise the granting of this licence.

For peer review only

Sexual and reproductive health service utilization of youth girls in rural Ethiopia: What are the roles of health extension workers?: Community based cross sectional study

Meskerem Jisso[1]*, Merga Belina Feyasa[2], Girmay Medhin[3,4], Tegene Legese Dadi[1], Yilkal Simachew[1], Bisrat Denberu[5], Mulusew Gerbaba Jebena [6], Yibeltal Kiflie Alemayehu[6,4] Alula Meresa Teklu[4]

Affiliation

1: Hawassa University College of medicine and health science, School of Public Health

2: Addis Ababa University, Department of statistics

3: Addis Ababa University, Aklilu Lemma Institute of Pathobiology

4: MERQ consultancy PLC

5: Federal minister of health, Ethiopia

6: Jimma University Institute of Health

***Corresponding author**

Meskerem Jisso

1: Hawassa University College of medicine and health science, School of public health

Email: Meskyj@gmail.com

For peer review only

Abstract

Objective: Little is known about the extent to which Health Extension Program (HEP) has played its role to increase service uptake among youth girls. This study aims to estimate the status of youth girls' SRH services utilization in rural Ethiopia and to examine the role of health extension workers (HEWs) in this regard.

Design: cross-sectional study.

Setting: community based study among all nine regions of Ethiopia.

Participants: nine hundred two youth girls age 15-24 years were included to this study.

Method: We used data from the national HEP assessment, collected from March to May 2019. Multilevel binary logistic regression was used to investigate the association between exposure to HEP and sexual and reproductive health (SRH) services utilization of youth girls and we reported adjusted odds ratio with corresponding 95% confidence interval (CI) as measures of the degree of associations.

Result: Only 19.18% [95% CI: 16.74, 21.89] of young girls used SRH service with significant regional variability (Intra-regional variability (ICC) =17.16 %; 95% CI: 6.30, 39.99). Exposure to HEP [AOR=3.13, 95%CI, (2.03, 4.85)], understanding availability of HEP service [AOR=3.06, 95%CI (1.75, 5.33)], and having good trust in HEWs [AOR=1.82, CI (1.07, 3.10)], and other socio-demographic factors were significantly associated with increased SRH services utilization.

Outcome: SRH service utilization

Conclusion: Although the overall SRH service utilization of youth girls in rural Ethiopia was very low, HEWs have a great contribution to improving service utilization of youth girls through strong health education provided during home-to-home visits, school visits, and at Health Posts (HPs). More investment on this line has the potential to improve service uptake among youth girls. Encouraging HEWs to build trust among this segment of the population and creating awareness of SRH related services is crucial to improve service uptake.

For peer review only - http://bmjopen.bmj.com/site/about/guidelines.xhtml

Strength and limitation of this study

- The study was done at country level, which is representative data.
- It did not assess the attitude of HEWs about SRH delivery
- Recall bias that might have underestimated the magnitude of SRH services.
- We couldn't assess the effect of cultural factors as a barrier for utilizing SRH service qualitatively.

Background

Youth between 15-24 years of age account for more than 1.2 billion people globally with 90% of them living in developing countries (1). In sub-Saharan Africa, this segment of the population constitutes 23% of the region's population (2). In Ethiopia, 34% of the total population is below the age of 25 years and majority of them (79% of males and 78% of youth girls) resides in rural parts(3,4) and the share of youth in Africa is expected to rise to 28% by the year 2040 (2).

Having healthy youth means investing in a family, society, and a nation as a whole. Hence, investing in their health has a potential to break the cycle of poverty through increased productivity, decreased health costs, and improved social capital (5). On the other hand, these groups of society are in great physical, physiological and emotional changes, which aggravate their vulnerability to sexual and reproductive health (SRH) problems (5). Globally one in 25 youth girls were at risk of early pregnancy in 2018, which is the leading cause of mortality (6). Three million youth girls undergo an unsafe abortion, 460,000 were newly infected with HIV and 333 million of them were infected with sexually transmitted infection (STI) (7). Similarly, in Ethiopia, , 0.4% of youths were infected by HIV/AIDS (8), and among university and college students 41.62% are engaged in risky sexual behavior (9).

Previous studies in Ethiopia showed that the SRH knowledge of youth girls and their SRH service utilization is very low, which might worsen their SRH problem (3). Only 37% of married youth girls utilize family planning (FP) services (10). On the other hand, one-third of married and 12% of unmarried young women have an unmet need for FP (10). Lack of SRH related information, poor perceptions about SRH, feeling of shame, fear of being seen by others,

For peer review only - http://bmjopen.bmj.com/site/about/guidelines.xhtml

restrictive cultural norms and inaccessibility to health services are among the underlying causes for this low service utilization (3,11).

Cognizant of these facts, the Ethiopian government launched a health extension program (HEP) in 2003 as a community-based program to increase quality and access to health services in order to prevent disease, promote healthy behaviors, and improve knowledge and services utilization (4,12). Youth reproductive health is one of the 18 HEP packages designed to be delivered at a grass-root level (12). Health extension workers (HEWs) are expected to educate the community on these packages specifically SRH related issues and provide different health services through a home-to-home visit, school visit, and at the health post (HP) level (13). Nonetheless, to date, to the best of our knowledge, there is limited evidence about the extent to which SRH services are utilized by youth girls (14). Having such evidence is useful to monitor the performance of HEP in addressing one of the health service uptakes by youth girls. This study aims to assess the level of SRH service utilization of youth girls in rural Ethiopia and to examine the role of HEPin this regard.

Methods

Study Context

During the data collection Ethiopia was administratively divide to nine regions and two city administration. Each region is hieratically divided into zones, districts, and then kebeles. A kebele is the smallest administrative unit expected to serve an average of 5,000 people and district is the lowest budget center of the government. More than 95% of the kebeles in the country have health posts Health posts are categorized under the primary level of Ethiopian health system tiers, constructed within each kebeles and staffed on average by two Health Extension Workers (HEWs). Each HEW are expected to serve from 1,500 up to 2,500 people and there are close to 40,000 HEWs in more than 17,000 health posts (HPs) in Ethiopia HEWs are high school graduates with additional one year of education that enables them to be engaged in health education, delivery of family planning methods and selected curative services. They are expected to spend 75% of their time in outreach service (home-to-home visit, school visit, and community campaign) and 25% on curative service at the HP. For the success of the HEP program, they are supervised at least weekly and monitored by the nearby primary health care

For peer review only - http://bmjopen.bmj.com/site/about/guidelines.xhtml

unit health professionals (15). Health education on SRH issues is supposed to be provided by HEWs both at health posts and at the household level. Services like delivery, selected FP methods (intrauterine devices, Norplant, and permanent FP), treatment for STI or HIV are provided at the health center or hospital level rather than at the HP level. In these conditions, HEWs are expected to refer the clients to the catchments health center after counseling them (12).

Study design, setting and data source

A cross-sectional national survey was conducted using multistage sampling method to evaluate the Ethiopian rural health extension program, and the detailed methods and sampling procedures are described elsewhere(16). This survey covered all the nine administrative regions of the country and data collection was conducted from March to May 2019. In total 62 districts were covered in the assessment. Within each district three kebeles were randomly selected and within each of the selected kebele 34 households were randomly selected from the total list of households in the kebele. After identifying the target household, young girls whose age is 15-24 years were eligible for interview: The details of the data collection procedures was described elsewhere(16).

Data collection for the HEP assessment

During the HEP program evaluation assessment, we used a structured questionnaire adapted from different literature(17–19). Interview administered field data collection was facilitated using Open Data Kit (ODK) application with youth girls at each kebele. The questionnaire was translated in to different local languages of Ethiopia and all study participants were interviewed by their local languages. Then the questionnaire was pre-tested before the actual data collection and language barrios and jargon words were corrected and also missed valuable variables were added to the questionnaire. Different techniques were used to minimize recall bias. Data collectors (both male and female) were recruited and received 10-day training on study objectives, data collection methods, ethical procedures, and data quality issues.

Participants for the current study

For peer review only - http://bmjopen.bmj.com/site/about/guidelines.xhtml

We extracted a total of 902 youth girls aged 15-24 years from a total of 6,364 rural households included in the HEP program evaluation assessment(20).

Measurements

Outcome variable

Youth sexual and reproductive health service utilization: This is a dichotomous outcome variable that refers to the status of youth girl in utilizing at least one of SRH services: (a) attending health education on SRH issue (during a home visit or at school or during HP visit) or (b) FP utilization (utilization of at least one modern FP method like IUCD, implants, injectable, condoms, pills). The variable assumes a value of “1” if a given youth girl utilized at least one of the SRH services and “0” if she did not utilize any.

Key Exposure variable

Exposure to HEW: This is the exposure variable that we used to investigate the role of HEP in enhancing SRH service utilization of youth girls. We measured this variable as follows: youth girls who meet HEW for any service at a home visit, school visit, or HP visits within one year period prior to data collection time were labeled as “exposed to HEW” or else they were labeled as “Not exposed”.

Other independent variable

Socio-demographic characteristics: age, educational status, marital status (categorized as never married and ever married (married, separated, and widowed)), source of information for FP, region, and wealth index of the household (categorized as lower, middle, and high).

Trust on HEW: This is a composite variable constructed from seven perception-related questions. Those youth girls who answered strongly agree or moderately agree to all seven perception-related questions were considered to have “good trust on HEWs” and those who answered strongly or moderately disagree were labeled as “not having trust on HEWs”

Understanding availability of SRH related HEP services at HP: Youth girls who know at least two SRH related HEP packages/services (health education, delivery, ANC, PNC, FP, HIV

For peer review only - http://bmjopen.bmj.com/site/about/guidelines.xhtml

test) were considered as having an "adequate understanding" and the other youth girls were considered as having "inadequate understanding".

Modern FP method knowledge: Youth girls who know at least one of modern FP methods (i.e. injectable, pills, Intrauterine device (IUD), condom (both male and female) and implants) were labeled as "knowledgeable about modern FP methods", and those who were not able to list any one of the four methods were considered as "not knowledgeable about modern FP methods".

Knowledge of HIV transmission: This is another composite variable that have used responses from nine questions. Youth girl who answered at least one correct answer from a total of nine ways of HIV transmission-related questions was labeled as having "knowledge of HIV transmission" and if she did not get correct answer for all the nine ways of HIV transmission-related questions, she was considered as "not knowledgeable about HIV transmission".

Statistical Analysis

We used Stata 14 for the analysis and reported frequencies, percentages, mean, and standard deviations as descriptive summary measures of key variables. We used multilevel binary logistic regression to model the odds of utilizing SRH services. Due to the multistage cluster sampling procedure, individual youths were nested within kebeles and then within regions. We examined the effect of the individual-level variables, and the region using a two-level logistic regression modeling. During analysis, the characteristics of youths were taken as individual level (level-1), and regions were taken as level-2. Region was considered as level two to understand regional variability which is useful for future intervention.

Based on this model, the intra-class correlation coefficient (ICC) was calculated to evaluate whether the variation in the odds of utilizing SRH services was primarily within or between regions. Both the crude odds ratio (cOR) and the adjusted odds ratio (aOR) with their corresponding 95% Confidence Interval (CI) were reported as measures of strength of association

Ethical consideration

For peer review only - http://bmjopen.bmj.com/site/about/guidelines.xhtml

189 The ethical letter was obtained from Ethiopian public health association (1613/820) and
190 informed consent was obtained from the participants.

191 Result

192 Socio-demographic characteristics

193 A total of 902 youth girls were interviewed, yielding 100% response rate. The mean age of the
194 respondents was 17.48 years (SD = ±2.27), 816 (80%) youth girls were in the age range of 15-19
195 years, one-fifth (18.82%) were illiterate, and 779 (86.36%) were never married (Table 1).

196 **Table 1: socio-demographic and household-level characteristics of youth girls in Ethiopia,**
197 **2020.**

Characteristics	Number	Percent
Age		
15-19	721	79.90
20-24	181	20.10
Educational status		
No formal education	170	18.85
Primary	514	56.98
Secondary and above	218	24.17
Marital Status		
Single	779	86.36
Ever married	123	13.64
Region		
Tigray	117	12.97
Afar	63	6.98
Amhara	169	18.74
Oromia	162	17.96
Somali	97	10.75
Benishangul Gumuz	50	5.54
SNNPR	150	16.63
Gambela	36	3.99
Harari	58	6.43
Source of information for FP		
HEW	186	23.4
Other health professionals	52	6.54
Other sources (TV, radio, friend, relatives, and printed materials)	557	70.06
Wealth index		
Lower	290	32.88
Middle	186	21.09

For peer review only - http://bmjopen.bmj.com/site/about/guidelines.xhtml

Higher	406	46.03

Note: FP: Family Planning; HEW; Health extension worker; SNNPR: South Nation Nationality People Region; TV: Television

Knowledge about SRH services and HEP related perception

Only one hundred seventeen (12.97%) youth girls know at least one method of FP and 97(10.75%) youth girls were knowledgeable about modes of HIV transmission and prevention. Six hundred fifty-one (76.77%) youth girls have a good perception about the HEW code of conduct, 602(71.92) have good trust in HEW and 732 (86.73%) have a good perception about HEP services. Three hundred forty-nine (38.69%) youth girls recommended an increase in the frequency of home visits (Table 2).

Table 2: Knowledge and perception of youth girls on sexual and reproductive health-related health extension package in Ethiopia, 2020.

Variables	Number	Percent
Knowledge of FP methods		
Knowledgeable	117	12.97
Not knowledgeable	785	87.03
Comprehensive knowledge about HIV		
Knowledgeable	97	10.75
Not knowledgeable	805	89.25
Have a good perception of the HEW code of conduct		
Strongly disagree	41	4.83
Moderately disagree	156	18.40
Moderately agree	490	57.78
Strongly agree	161	18.99
Have good trust in HEW		
Strongly disagree	36	4.30
Moderately disagree	199	23.78
Moderately agree	438	52.33
Strongly agree	164	19.59

For peer review only

For peer review only - http://bmjopen.bmj.com/site/about/guidelines.xhtml

Have good perception on SRH service delivery by HEW		
Strongly disagree	31	3.67
Moderately disagree	81	9.60
Moderately agree	442	52.37
Strongly agree	290	34.36
Which SRH related HEP services do you know?		
Health education	633	71.08
Family planning	540	60.39
Antenatal care	534	58.82
Delivery service	269	30.49
Postnatal care	359	40.39
Allocation of time for HP and home visit		
Increase the frequency of home visit	349	38.69
Increasing the time, they spend at HP	131	14.52
Increase both of them	347	38.47
Keep the current allocation of time	75	8.31

Utilization of SRH service

National level SRH service utilization of youth girls is 19.18% (95% CI [16.74, 21.89]. Among the 244 youth girls that live in pastoralist areas only 29 (11.90%) utilized SRH services Similarly, among 658 youth girls that live in agrarian areas, 144(21.9%) utilized SRH services. There is a significant amount of variation in the status of SRH utilization across the region accounting 17.16% of the total variability (ICC=17.16 % ; 95% CI: 6.30, 39.99).

SRH service use among youth girls was significantly higher if they have exposure to HEP through HEWs' home to home visits, school visits, or during HP visits (70.5%) as compared to those who do not have such exposure (37.6%).

Determinants of SRH services utilization use among youth girls

Youth girls who were exposed to HEW were three times more likely to utilize SRH services [AOR=3.13, 95% CI, (2.03, 4.85)].

Other factors that are associated with increased SRH service utilization are understanding that SRH related services are available at HPs [aOR=3.05, 95%CI (1.75, 5.33)], having good trust in HEW [aOR=1.82, CI (1.07, 3.10)], being in the age range of 20-24 years [aOR=2.93, CI (1.86,

For peer review only - http://bmjopen.bmj.com/site/about/guidelines.xhtml

4.63)], and being ever married [aOR=6.54, CI (3.82, 11.19)] compared to their counterparts (Table 3).

Table 3: Individual and region level factors associated with youth SRH service utilization from HEP in Ethiopia, 2020.

Variables	Utilization of SRH		COR (95% CI)	AOR (95% CI)
	No	Yes		
Exposure to HEW				
Not exposed	62.41	29.48	1.00	1.00
Exposed	37.59	70.52	3.61(2.47, 5.26)	3.13(2.03, 4.85)
Understanding HEP service availability				
Inadequate	91.50	8.50	1.00	1.00
Adequate	75.66	24.34	3.53 (2.19, 5.69)	3.05 (1.75, 5.33)
Trust on HEW				
Good	87.66	12.34	2.17(1.38, 3.42)	1.82(1.07, 3.10)
Poor	77.08	22.92	1.00	1.00
Age				
15-19	86.13	13.87	1.00	1.00
20-24	59.67	40.33	4.44 (3.01, 6.55)	2.93 (1.86, 4.63)
Marital status				
Ever Married	86.39	13.61	8.24 (5.17, 13.13)	6.54 (3.82, 11.19)
Never married	45.53	54.47	1.00	1.00

Note: HEW: Health Extension Worker, HEP: Health Extension Program, SRH: Sexual and Reproductive Health

Discussion

In this study, the status of SRH service utilization of youth girls was very low and it was increased when they were exposed to HEP through health education provided by HEW. Exposure to HEWs, understanding HEP service availability, and having trust in HEW were significantly associated with increased SRH service utilization among youth girls. While older age and being ever married were significantly associated with increased SRH service utilization, educational status of youth girls, wealth index of the household, perception about SRH service delivery by HEW, and perception of code of conduct of HEWs were not significantly associated with SRH service utilization of youth girls.

For peer review only

For peer review only - http://bmjopen.bmj.com/site/about/guidelines.xhtml

SRH service utilization of youth girls was relatively lower compared to the findings of previous studies in Ethiopia (19,21–23). One possible reason for the discrepancy might be the difference in the study settings; participants for the current study are from rural areas where resources and access to services are very limited(23). This low utilization might also be linked to a low level of knowledge among youth girls implying that focus given to youth girls in rural areas by HEWs is likely to be minimal(24). This assumption is also supported by the low percentage of youth girls in the current study having exposure to HEP services during a home-to-home visit as well as during their schooling. Encouraging HEWs to give due attention to youth girls in rural areas might be one of the strategies that should be followed to increase their service uptake of youth girls in rural Ethiopia that is home of the significant majority of the population(12).

One of the HEP packages was designed to increase youth SRH service utilization through health education during a home-to-home visit, through school visit, and during HP based service provision (24,25). In line with this, the current finding showed that HEWs had a great role in increasing the SRH service utilization of youth girls. Whenever the HEWs meet youth girls in their home, at school, or at HP the odds of youth girl's SRH service utilization was increased(12). This is a general indication that strengthening the role of the HEWs in implementing HEP strategies can improve SRH service utilization of youth girls.

An adequate understanding of available components of HEP is significantly associated with increased service utilization among youth girls. Among different components of SRH related HEP packages, most of the respondents recalled health education and family planning services. This may reflect the differential focus given to FP education by HEWs during any service delivery. Studies in Ethiopia and Uganda showed that knowing the place of SRH service increases service utilization(25,26). One of the global quality standard measures also indicates that someone is knowledgeable about their health only if they know where and when to get those services (27). This is based on the assumption that the more they know about the available service the more likely for them to utilize the service in the nearby HP.

Confidentiality is one of the major ethical codes in medicine and SRH service utilization require similar level of confidentiality because of its sensitivity (11,28). Youth girls are very worried, and they do not want to be seen by their parents, friends, or other people they know (relatives,

For peer review only - http://bmjopen.bmj.com/site/about/guidelines.xhtml

teachers, or neighbors) while receiving SRH services. This knowledge makes them vulnerable to stigma and mortification(29). The International Federation of Gynecology and Obstetrics (FIGO) recognizes that health professionals are obligated to adhere to their patients' confidentiality (30). In the current study youth girls who have a good trust in HEW were more likely to utilize SRH service than their counterparts. Other studies also reported that increasing the privacy and confidentiality of youths improved their service utilization (31,32). Young girls should be provided confidential and flexible service in youth friendly manner which can increase their trust and service utilization(33,34).

In line with previous Ethiopian study, conducted at Baher Dar, youth girls at the age of 20-24 years were more likely to utilize SRH services than 15-19 age girls (31). Sexual and reproductive health demand increases with age due to a change in marital status that could lead to birth spacing, and educational status that could increase their ability to understand the risk of exposure to sexual practice (8).

Although the findings of the Ethiopian Demographic Health Survey showed that many youth women are engaged in sexual intercourse before marriage, the issue of SRH is culturally sensitive and socially unacceptable for unmarried girls which may decrease service utilization among youth girls (8). In agreement with this, unmarried youth girls in the current study were less likely to utilize SRH services from HEP. The negative attitude of health workers as a result of lack of training was provided as a justification for similar findings in a previous Ethiopian study (35).

Conclusion

This study demonstrates that SRH service utilization of youth girls in rural Ethiopia is very low. Our findings also indicate that HEWs have a significant contribution to improving SRH service utilization of youth girls through health education during home-to-home visits, school visits, and at HPs. However, more effort might be required to significantly increase the exposure of youth girls to HEP services to increase service uptake. Encouraging youth girls to build trust in HEWs and to make youth girls know what SRH related services are available at the HP is the first step to improve service uptake. HEWs need to give special focus on younger youth girls and never married youth girls to increase their service uptake.

For peer review only

For peer review only - http://bmjopen.bmj.com/site/about/guidelines.xhtml

Acknowledgment

We are very thankful for MERQ consultancy for granting us to access this data source for this study. We acknowledge Ms. Saron who thoroughly edited this manuscript for language usage, spelling and grammar.

Competing interests: The authors have declared that they do not have any competing interests exist.

Funding: The study was conducted by MERQ Consultancy PLC as part of a national assessment on the Health Extension Program of Ethiopia. The national assessment was financed through a grant from the Bill & Melinda Gates Foundation (INV010174). The funder provided financial support to MERQ that covered professional fees for leaders but did not have any additional role in the study design, data collection and analysis, decision to publish, or preparation of the manuscript. The specific roles of these authors are articulated in the "author contributions" section.

Author Contributions: AMT YK GM TLD contributed to the conceptualization, designing and conduct of the national HEP assessment, MGJ TLD MJ conceptualized the idea coved within this paper, MJ drafted the MS, MB lead the data analysis and involved in drafting of the MS. GM MGJ TLD supervised the write-up of the draft, all coauthors critically reviewed the drat MS and contributed intellectual inputs to improve the content of the MS and endorsed the final version of the MS to be submitted for possible publication.

Data availability: questionnaire and STATA do file

References

1. UN. Youth and the 2030 agenda for sustainable development. 2019.

2. United Nations. World Population Monitoring Adolescent and Youth Department of Economic and Social Affairs Population Division [Internet]. United Nations. 2012. Available from: http://www.un.org/en/development/desa/population/publications/pdf/fertility/12_66976_a

For peer review only - http://bmjopen.bmj.com/site/about/guidelines.xhtml

dolescents_and_youth.pdf

3. FMOH. Adolescence And Youth Health(AYH) Case Team | FMOH [Internet]. 2016 [cited 2021 Mar 23]. Available from: http://www.moh.gov.et/index.php/am/AYH

4. FMOH. Health Sector Development Program IV In line with GTP [Internet]. 2016. 10–137 p. Available from: https://www.ccghr.ca/wp-content/uploads/2013/11/healthsectordevelopmentprogram.pdf%0Ahttp://tucghe.org/index.php/component/search/?searchword=HSDP ethiopia&searchphrase=all&Itemid=435

5. UNFPA. The Case for Investing in Young People as part of a National Poverty Reduction Strategy [Internet]. 2005. Available from: http://www.unfpa.org/webdav/site/global/shared/documents/publications/2005/case_youngpeople_eng.pdf

6. WHO. World health statistics overview:monitoring health for the SDGs. 2019.

7. Dehne KL, Riedner G. Sexually transmitted infections among adolescents: The need for adequate health services. Reprod Health Matters. 2001;9(17):170–83.

8. Central Statistical Agency [Ethiopia], ICF International. Ethiopia Demographic and Health Survey 2011. Ethiop Water Sect Dev Progr [Internet]. 2012;1–452. Available from: https://www.usaid.gov/sites/default/files/documents/1860/Ethiopia DHS 2016 KIR - Final 10-17-2016.pdf

9. Amare T, Yeneabat T, Amare Y. A Systematic Review and Meta-Analysis of Epidemiology of Risky Sexual Behaviors in College and University Students in Ethiopia , 2018. Hindawi. 2019;10(1155):8.

10. CSA. Ethiopia Mini Demographic and Health Survey. 2019.

11. Birhanu Z, Tushune K, Jebena MG. Sexual and Reproductive Health Services Use, Perceptions, and Barriers among Young People in Southwest Oromia, Ethiopia. Ethiop J Health Sci. 2018;28(1):37–48.

12. FMOH. Ethiopia second Generation Health Extension Program. 2015.

For peer review only

For peer review only - http://bmjopen.bmj.com/site/about/guidelines.xhtml

13. Abebe Alebachew WC. Improving health system efficiency: Ethiopia human resources for health reforms. 2015.

14. Kemal M. Federal Democratic Republic of Ethiopia Ministry of Health Adolescent Federal Democratic Republic of Ethiopia Ministry of Health Adolescent Reproductive Health Extension Package. researchGate. 2003;(April).

15. FMOH. Federal democratic republic of Ethiopia ministry of health, 2015 Health sector transformation Plan (HSTP). 2015.

16. Merq conseltancy PPLC. The national assessment of the Ethiopian health evidence brief for action evidence brief. 2020;

17. Yitayal M., Et.al. Health extension program factors, frequency of household visits and being model households, improved utilization of basic health services in Ethiopia. BMC Health Serv Res [Internet]. 2014;14:156. Available from: http://ovidsp.ovid.com/ovidweb.cgi?T=JS&PAGE=reference&D=emed12&NEWS=N&AN=24708661

18. Central Statistical Agency (CSA). Ethiopian Demographic Health survey. Vol. Volume 8. 2017.

19. Alemu AA, Assefa S. Reproductive health knowledge and services utilization among rural adolescents in east Gojjam zone , Ethiopia : a community-based cross-sectional study. BMC Health Serv Res. 2014;14(138):1–11.

20. Alula M. Teklu, Yibeltal K. Alemayehu, Girmay Medhin et al. National Assessment of Ethiopian Health Extension Program Addis Ababa, Ethiopia: MERQ Consultancy PLC. 2020.

21. Binu W, Marama T, Gerbaba M, Sinaga M. Sexual and reproductive health services utilization and associated factors among secondary school students in Nekemte town, Ethiopia. Reprod Health. 2018;15(1):1–10.

22. Gebreyesus H, Teweldemedhin M, Mamo A. Determinants of reproductive health services

For peer review only

For peer review only - http://bmjopen.bmj.com/site/about/guidelines.xhtml

utilization among rural female adolescents in Asgede-Tsimbla district Northern Ethiopia: A community based cross-sectional study. Reprod Health. 2019;16(1):1–10.

23. Ayehu A, Kassaw T HG. Level of young people sexual and reproductive health service utilization and its associated factors among young people in Awabel District, Northwest Ethiopia. PLoS One. 2016;11(3):1–11.

24. Yitayal M, Berhane Y, Worku A, Kebede Y. Health extension program factors , frequency of household visits and being model households , improved utilization of basic health services in Ethiopia. BMC Health Serv Res [Internet]. 2014;14(1):1–9. Available from: BMC Health Services Research

25. Na M, Jennings L, Talegawkar SA, Ahmed S. Association between women's empowerment and infant and child feeding practices in sub-Saharan Africa: An analysis of Demographic and Health Surveys. Vol. 18, Public Health Nutrition. 2015. p. 3155–65.

26. Nair M, Phil D, Baltag V, Ph D, Bose K, Ph D, et al. Improving the Quality of Health Care Services for Adolescents , Globally : A Standards-Driven Approach. J Adolesc Heal [Internet]. 2015;57(3):288–98. Available from: http://dx.doi.org/10.1016/j.jadohealth.2015.05.011

27. FIGO. International Federation of Gynecology and Obstetrics. Ethical Issues in Obstetrics and Gynecology. FIGO Committee for the Study of Ethical Aspects of Human Reproduction and Women's Health. London. 2012. 1–148 p.

28. ACHE Code of Ethics | American College of Healthcare Executives [Internet]. [cited 2021 Mar 24]. Available from: https://www.ache.org/about-ache/our-story/our-commitments/ethics/ache-code-of-ethics

29. Adolescent and Youth-Friendly Services | The Challenge Initiative [Internet]. [cited 2021 Jul 3]. Available from: https://tciurbanhealth.org/courses/adolescent-youth-sexual-reproductive-health-services-supply/lessons/adolescent-and-youth-friendly-services-3/

30. Denno DM, Hoopes AJ, Chandra-Mouli V. Effective strategies to provide adolescent sexual and reproductive health services and to increase demand and community support. J

For peer review only

For peer review only - http://bmjopen.bmj.com/site/about/guidelines.xhtml

404 Adolesc Heal [Internet]. 2015;56(1):S22–41. Available from:
405 http://dx.doi.org/10.1016/j.jadohealth.2014.09.012

406 31. Abebe M, Awoke W. Utilization of Youth Reproductive Health Services and Associated
407 Factors among High School Students in Bahir Dar, Amhara Regional State, Ethiopia.
408 Open J Epidemiol. 2014;04(02):69–75.

409 32. Feleke SA, Koye DN, Demssie AF, Mengesha ZB. Reproductive health service utilization
410 and associated factors among adolescents (15-19 years old) in Gondar town, Northwest
411 Ethiopia. BMC Health Serv Res. 2013;13(1):1–10.

412 33. Haile B, Shegaze M, Feleke T, Glagn M, Andarge E. Disparities in utilization of sexual
413 and reproductive health services among high school adolescents from youth friendly
414 service implemented and non-implemented areas of Southern Ethiopia. Arch Public Heal
415 [Internet]. 2020 Dec 1 [cited 2021 Mar 23];78(1):126. Available from:
416 https://archpublichealth.biomedcentral.com/articles/10.1186/s13690-020-00508-w

417 34. Cherie A, Yemane Berhane and. Knowledge of Sexually Transmitted Infections and
418 Barriers to Seeking Health Services among High School Adolescents in Addis Ababa.
419 Ethiop J AIDS Clin Res. 2012;2012:153.

420 35. Tilahun M, Mengistie B, Egata G, Reda AA. Health workers ' attitudes toward sexual and
421 reproductive health services for unmarried adolescents in Ethiopia. Reprod Health.
422 2012;9(19):1–7.

423

424

For peer review only - http://bmjopen.bmj.com/site/about/guidelines.xhtml

STROBE Statement—Checklist of items that should be included in reports of ***cross-sectional studies***

	Item No	Recommendation	Page No
**Title and abstract**	1	(*a*) Indicate the study's design with a commonly used term in the title or the abstract	1
		(*b*) Provide in the abstract an informative and balanced summary of what was done and what was found	2
**Introduction**			
Background/rationale	2	Explain the scientific background and rationale for the investigation being reported	3&4
Objectives	3	State specific objectives, including any prespecified hypotheses	4
**Methods**			
Study design	4	Present key elements of study design early in the paper	5
Setting	5	Describe the setting, locations, and relevant dates, including periods of recruitment, exposure, follow-up, and data collection	4 &5
Participants	6	(*a*) Give the eligibility criteria, and the sources and methods of selection of participants	6
Variables	7	Clearly define all outcomes, exposures, predictors, potential confounders, and effect modifiers. Give diagnostic criteria, if applicable	6&7
Data sources/ measurement	8*	For each variable of interest, give sources of data and details of methods of assessment (measurement). Describe comparability of assessment methods if there is more than one group	*5*
Bias	9	Describe any efforts to address potential sources of bias	5
Study size	10	Explain how the study size was arrived at	6
Quantitative variables	11	Explain how quantitative variables were handled in the analyses. If applicable, describe which groupings were chosen and why	6&7
Statistical methods	12	(*a*) Describe all statistical methods, including those used to control for confounding	7
		(*b*) Describe any methods used to examine subgroups and interactions	7
		(*c*) Explain how missing data were addressed	7
		(*d*) If applicable, describe analytical methods taking account of sampling strategy	NA
		(*e*) Describe any sensitivity analyses	NA
**Results**			
Participants	13*	(a) Report numbers of individuals at each stage of study—eg numbers potentially eligible, examined for eligibility, confirmed eligible, included in the study, completing follow-up, and analysed	8
		(b) Give reasons for non-participation at each stage	Non
		(c) Consider use of a flow diagram	8
Descriptive data	14*	(a) Give characteristics of study participants (eg demographic, clinical, social) and information on exposures and potential confounders	8
		(b) Indicate number of participants with missing data for each variable of interest	8
Outcome data	15*	Report numbers of outcome events or summary measures	10
Main results	16	(*a*) Give unadjusted estimates and, if applicable, confounder-adjusted estimates and their precision (eg, 95% confidence interval). Make clear which confounders were adjusted for and why they were included	10

For peer review only

For peer review only - http://bmjopen.bmj.com/site/about/guidelines.xhtml

		(*b*) Report category boundaries when continuous variables were categorized	10,11
		(*c*) If relevant, consider translating estimates of relative risk into absolute risk for a meaningful time period	NA
Other analyses	17	Report other analyses done—eg analyses of subgroups and interactions, and sensitivity analyses	NA
**Discussion**			
Key results	18	Summarise key results with reference to study objectives	11
Limitations	19	Discuss limitations of the study, taking into account sources of potential bias or imprecision. Discuss both direction and magnitude of any potential bias	11-13
Interpretation	20	Give a cautious overall interpretation of results considering objectives, limitations, multiplicity of analyses, results from similar studies, and other relevant evidence	11-13
Generalisability	21	Discuss the generalisability (external validity) of the study results	11-13
**Other information**			
Funding	22	Give the source of funding and the role of the funders for the present study and, if applicable, for the original study on which the present article is based	14

*Give information separately for exposed and unexposed groups.

Note: An Explanation and Elaboration article discusses each checklist item and gives methodological background and published examples of transparent reporting. The STROBE checklist is best used in conjunction with this article (freely available on the Web sites of PLoS Medicine at http://www.plosmedicine.org/, Annals of Internal Medicine at http://www.annals.org/, and Epidemiology at http://www.epidem.com/). Information on the STROBE Initiative is available at www.strobe-statement.org.

For peer review only

For peer review only - http://bmjopen.bmj.com/site/about/guidelines.xhtml

BMJ Open

Sexual and reproductive health service utilization of young girls in rural Ethiopia: What are the roles of health extension workers?: Community based cross-sectional study

Journal:	*BMJ Open*
Manuscript ID	bmjopen-2021-056639.R1
Article Type:	Original research
Date Submitted by the Author:	01-Jun-2022
Complete List of Authors:	Jisso, Meskerem ; Hawassa University, Public health<br>Feyasa, Merga; Addis Ababa University College of Natural Sciences, Statistics<br>Medhin, Girmay; Addis Ababa University, Aklilu Lemma Institute of Pathology<br>Dadi, Tegene ; Hawassa University College of Medicine and Health Sciences, School of Public health<br>Simachew, Yilkal; Hawassa University College of Medicine and Health Sciences, School of public health<br>Denberu, Bisrat; federal ministry of health<br>Jebena, Mulusew; Jimma University College of Public Health and Medical Sciences<br>Alemayehun, Yibeltal ; MERQ Consultancy PLC<br>Teklu, Alula M.; MERQ Consultancy PLC ; MERQ PLC LLC, research
**Primary Subject Heading**:	Sexual health
Secondary Subject Heading:	Sexual health, Public health
Keywords:	REPRODUCTIVE MEDICINE, PUBLIC HEALTH, SEXUAL MEDICINE, EPIDEMIOLOGY, EDUCATION & TRAINING (see Medical Education & Training), STATISTICS & RESEARCH METHODS

SCHOLARONE™
Manuscripts

I, the Submitting Author has the right to grant and does grant on behalf of all authors of the Work (as defined in the below author licence), an exclusive licence and/or a non-exclusive licence for contributions from authors who are: i) UK Crown employees; ii) where BMJ has agreed a CC-BY licence shall apply, and/or iii) in accordance with the terms applicable for US Federal Government officers or employees acting as part of their official duties; on a worldwide, perpetual, irrevocable, royalty-free basis to BMJ Publishing Group Ltd ("BMJ") its licensees and where the relevant Journal is co-owned by BMJ to the co-owners of the Journal, to publish the Work in this journal and any other BMJ products and to exploit all rights, as set out in our licence.

The Submitting Author accepts and understands that any supply made under these terms is made by BMJ to the Submitting Author unless you are acting as an employee on behalf of your employer or a postgraduate student of an affiliated institution which is paying any applicable article publishing charge ("APC") for Open Access articles. Where the Submitting Author wishes to make the Work available on an Open Access basis (and intends to pay the relevant APC), the terms of reuse of such Open Access shall be governed by a Creative Commons licence – details of these licences and which Creative Commons licence will apply to this Work are set out in our licence referred to above.

Other than as permitted in any relevant BMJ Author's Self Archiving Policies, I confirm this Work has not been accepted for publication elsewhere, is not being considered for publication elsewhere and does not duplicate material already published. I confirm all authors consent to publication of this Work and authorise the granting of this licence.

For peer review only

Sexual and reproductive health service utilization of young girls in rural Ethiopia: What are the roles of health extension workers?: Community based cross sectional study

Meskerem Jisso[1]*, Merga Belina Feyasa[2], Girmay Medhin[3,4], Tegene Legese Dadi[1], Yilkal Simachew[1], Bisrat Denberu[5], Mulusew Gerbaba Jebena [6], Yibeltal Kiflie Alemayehu[6,4] Alula Meresa Teklu[4]

Affiliation

1: Hawassa University College of medicine and health science, School of Public Health

2: Addis Ababa University, Department of statistics

3: Addis Ababa University, Aklilu Lemma Institute of Pathobiology

4: MERQ consultancy PLC

5: Federal minister of health, Ethiopia

6: Jimma University Institute of Health

***Corresponding author**

Meskerem Jisso

1: Hawassa University College of medicine and health science, School of public health

Email: Meskyj@gmail.com

For peer review only

For peer review only - http://bmjopen.bmj.com/site/about/guidelines.xhtml

Abstract

Objective: Little is known about the extent to which Health Extension Program (HEP) has played its role to increase service uptake among young girls. This study aims to estimate the status of young girls' SRH services utilization in rural Ethiopia and to examine the role of health extension workers (HEWs) in this regard.

Design: A cross-sectional study.

Setting: A community-based study among all nine regions of Ethiopia.

Participants: nine hundred and two young girls age 15-24 years were included to this study.

Method: We used data from the national HEP assessment, collected from March to May 2019. Multilevel binary logistic regression was used to investigate the association between exposure to HEP and sexual and reproductive health (SRH) services utilization of young girls and we reported adjusted odds ratio with corresponding 95% confidence interval (CI) as measures of the degree of associations.

Result: Only 19.18% [95% CI (16.74, 21.89)] of young girls used SRH services with significant regional variability (Intra-regional variability [(ICC) =17.16 %; 95% CI (6.30, 39.99)]. Exposure to HEP [aOR=3.13, 95%CI, (2.03, 4.85)], knowing about the availability of HEP services [aOR=3.06, 95%CI (1.75, 5.33)], and having good trust in HEWs [AOR=1.82, CI (1.07, 3.10)], and other socio-demographic factors were significantly associated with increased SRH services utilization.

Outcome: SRH service utilization

Conclusion: Although the overall SRH service utilization of young girls in rural Ethiopia was very low, HEWs have a great contribution to improving service utilization of young girls through strong health education provided during home visits, school visits, and at Health Posts. More investment along this line has the potential to improve service uptake among young girls. Encouraging HEWs to build trust among this segment of the population and creating awareness of SRH related services is crucial to improve service uptake.

For peer review only - http://bmjopen.bmj.com/site/about/guidelines.xhtml

Strength and limitation of this study

- The study was done at country level, which is representative data.
- It did not assess the attitude of HEWs about SRH delivery
- Recall bias that might have underestimated the magnitude of SRH services.
- We couldn't assess the effect of cultural factors as a barrier for utilizing SRH service qualitatively.

Background

Youth between 15-24 years of age account for more than 1.2 billion people globally with 90% of them living in developing countries (1). In sub-Saharan Africa, this segment of the population constitutes 23% of the region's population (2). In Ethiopia, 34% of the total population is below the age of 25 years and the majority of them (79% of males and 78% of females) resides in rural areas (3). The number of young people in least developed countries is expected to rise from 207 million in 2019 to 336 million by the year 2050 (4).

Having healthy youth leads to improved outcomes for a family, society, and a nation as a whole. Hence, investing in their health has a potential to break the cycle of poverty through increased productivity, decreased health costs, and improved social capital (5). Yet young girls are undergoing great physical, physiological and emotional changes, which aggravate their vulnerability to sexual and reproductive health (SRH) problems (5). Globally, in 2018, one in 25 young girls were at risk of early pregnancy, which is the leading cause of mortality among young girls (6). Three million young girls undergo an unsafe abortion, 460,000 were newly infected with HIV and 333 million of them were infected with a sexually transmitted infection (STI) (7) in the world . Similarly, in Ethiopia by 2016, 0.4% of youths were infected by HIV/AIDS (8), and among university and college students based on a 2019 study 41.62% are engaged in risky sexual behavior (9).

Previous studies in Ethiopia showed that the SRH knowledge of young girls and their SRH service utilization is very low, which might worsen their SRH problems (3). A survey conducted in 2019 showed that only 37% of married young girls utilize family planning (FP) services (10).

For peer review only

For peer review only - http://bmjopen.bmj.com/site/about/guidelines.xhtml

On the other hand, one-third of married and 12% of unmarried young girls have an unmet need for FP (10). Lack of SRH related information, poor perceptions about SRH, feeling of shame, fear of being seen by others, while they are utilizing the SRH services, restrictive cultural norms and inaccessibility to health services are among the underlying causes for this low service utilization (3,11).

Cognizant of these facts, the Ethiopian government launched a health extension program (HEP) in 2003 as a community-based program to increase quality of and access to health services in order to prevent disease, promote healthy behaviors, and improve knowledge and service utilization (12,13). Youth reproductive health is one of the 18 HEP packages designed to be delivered at a grass-root level (13). HEWs are expected to educate the community on these packages, and specifically on SRH related issues, and provide different health services through a home visits, school visits, and at health posts (HPs) (14). Nonetheless, to date, to the best of our knowledge, there is limited evidence about the extent to which SRH services are utilized by young girls (15). Having such evidence is useful to monitor the performance of HEP in addressing one of the health service uptakes by young girls. This study aims to assess the level of SRH service utilization of young girls in rural Ethiopia and to examine the role of HEP in this regard.

Methods

Study Context

During the data collection Ethiopia was administratively divided into nine regions and two city administrations. Each region is hierarchically divided into zones, districts, and then kebeles. A kebele is the smallest administrative unit expected to serve an average of 5,000 people, and a district is the lowest budget center of the government. More than 95% of the kebeles in the country have HPs. HPs are categorized under the primary level of Ethiopian health system tiers, constructed within each kebele and staffed on average by two HEWs. Each HEW is expected to serve from 1,500 up to 2,500 people and there are close to 40,000 HEWs in more than 17,000 HPs in Ethiopia.

For peer review only - http://bmjopen.bmj.com/site/about/guidelines.xhtml

HEWs are high school graduates with additional one year of education that enables them to be engaged in health education, and delivery of FP methods and selected curative services. They are expected to spend 75% of their time in outreach service (home visits, school visits, and community campaigns) and 25% on curative services at the HP. For the success of the HEP program, HEWs are supervised at least weekly and monitored by the nearby primary health care unit health professionals (16). Health education on SRH issues is supposed to be provided by HEWs both at health posts and at the household level. Services like Skilled birth, selected FP methods (intrauterine devices, Norplant, and permanent FP), and treatment for STI or HIV are provided at the health center or hospital level rather than at the HP level. In these cases, HEWs are expected to refer the clients to the catchment health center after counseling them (13).

Study design, setting and data source

A cross-sectional national survey was conducted using a multistage sampling method to evaluate the Ethiopian rural HEP, and for this study data was extracted from this survey. The survey covered all the nine administrative regions of the country and data collection was conducted from March to May 2019. In total, 62 (both agrarian and pastoralist regions) districts were covered in the assessment by using appropriate methodological flow. Within each district three kebeles were randomly selected and then within each selected kebeles, 34 households were randomly selected from the total list of households in the kebele. A total of 902 young girls between the age of 15-24 years from a total of 6,324 rural households were included in the HEP program evaluation assessment and we have used these data for analysis. The detailed methods and sampling procedures are described elsewhere (17).

Data collection for the HEP assessment

During the HEP program evaluation assessment, we used a structured questionnaire adapted from different literature (18–20). Interview administered field data collection was facilitated using Open Data Kit (ODK) application with young girls at each kebele. The questionnaire was translated in to different local languages of Ethiopia and all study participants were interviewed in their local languages. The questionnaire was pre-tested before the actual data collection and language barriers and jargon words were corrected and also missed valuable variables were added to the questionnaire. Different techniques were used to minimize recall bias. Data

For peer review only - http://bmjopen.bmj.com/site/about/guidelines.xhtml

collectors (both male and female) were recruited and received 10-day training on study objectives, data collection methods, ethical procedures, and data quality issues.

Patient and Public Involvement

No patient involved

Measurements

Outcome variable

Young girls sexual and reproductive health service utilization: This is a dichotomous outcome variable that refers to the status of young girl in utilizing at least one of SRH services: (a) attending health education on SRH issue (during a home visit or at school or during HP visit) or (b) FP utilization (utilization of at least one modern FP method like IUCD, implants, injectable, condoms, pills). The variable assumes a value of "1" if a given young girl utilized at least one of the SRH services and "0" if she did not utilize any.

Key Exposure variable

Exposure to HEW: This is the exposure variable that we used to investigate the role of HEP in enhancing SRH service utilization of young girls. We measured this variable as follows: young girls who meet HEW for any service at a home visit, school visit, or HP visits within a one-year period prior to data collection time were labeled as "exposed to HEW" or else they were labeled as "Not exposed".

Other independent variable

Socio-demographic characteristics: age, educational status, marital status (categorized as never married and ever married (married, separated, and widowed)), source of information for FP, region, and wealth index of the household (categorized as lower, middle, and high).

Trust in HEW: This is a composite variable constructed from seven perception-related questions, intended to measure a young woman's attitude toward HEWs. Those young girls who answered strongly agree or moderately agree to all seven perception-related questions were

For peer review only - http://bmjopen.bmj.com/site/about/guidelines.xhtml

considered to have "good trust in HEWs" and those who answered strongly disagree or moderately disagree were labeled as "poor trust in HEWs "

Knowing availability of SRH-related HEP services at HP : Young girls who know at least two SRH related HEP packages/services (health education, delivery, antenatal care (ANC), postnatal care (PNC), FP, HIV test) were considered as having an "adequate knowledge" and the other young girls were considered as having "inadequate knowledge".

Modern FP method knowledge: Young girls who know at least one modern FP method (i.e. injectable, pills, Intrauterine device (IUD), condom (both male and female) and implants) were labeled as "knowledgeable about modern FP methods", and those who were not able to list any one of the five methods were considered as "not knowledgeable about modern FP methods".

Knowledge of HIV transmission: This is another composite variable that used responses from nine questions. A young girl who gave at least one correct answer from a total of nine HIV transmission-related questions was labeled as having "knowledge of HIV transmission" and if she did not give a correct answer for any of the nine HIV transmission-related questions, she was considered as "not knowledgeable about HIV transmission"

Statistical Analysis

We used Stata 14 for the analysis and reported frequencies, percentages, mean, and standard deviations as descriptive summary measures of key variables. We used multilevel binary logistic regression to model the odds of utilizing SRH services. Due to the multistage cluster sampling procedure, individual young girls were nested within kebeles and then within regions. We examined the effect of the individual-level variables, and the region-level variables using two-level logistic regression modeling. During analysis, the characteristics of young girls were taken as individual level (level-1), and regions were taken as level-2. Region was considered as level two to understand regional variability which is useful for future intervention.

Based on this model, the intra-class correlation coefficient (ICC) was calculated to evaluate whether the variation in the odds of utilizing SRH services was primarily within or between regions. Both the crude odds ratio (cOR) and the adjusted odds ratio (aOR) with their

For peer review only - http://bmjopen.bmj.com/site/about/guidelines.xhtml

corresponding 95% Confidence Interval (CI) were reported as measures of strength of association

Ethical consideration

The ethical letter of approval was obtained from Ethiopian public health association (1613/820) and informed consent was obtained from the participants.

Results

Socio-demographic characteristics

A total of 902 young girls were interviewed, yielding 100% response rate. The mean age of the respondents was 17.48 years (SD = ±2.27), 816 (80%) young girls were in the age range of 15-19 years, 170 (18.82%) had no formal education, and 779 (86.36%) were never married (Table 1).

Table 1: socio-demographic and household-level characteristics of young girls in Ethiopia, 2020.

Characteristics	Number	Percent
Age		
15-19	721	79.90
20-24	181	20.10
Educational status		
No formal education	170	18.85
Primary	514	56.98
Secondary and above	218	24.17
Marital Status		
Single	779	86.36
Ever married	123	13.64
Region		
Tigray	117	12.97
Afar	63	6.98
Amhara	169	18.74
Oromia	162	17.96
Somali	97	10.75
Benishangul Gumuz	50	5.54
SNNPR	150	16.63
Gambela	36	3.99
Harari	58	6.43
Source of information for FP		
HEW	186	23.4

For peer review only - http://bmjopen.bmj.com/site/about/guidelines.xhtml

Other health professionals	52	6.54
Other sources (TV, radio, friend, relatives, and printed materials)	557	70.06
Wealth index		
Lower	290	32.88
Middle	186	21.09
Higher	406	46.03

Note: FP: Family Planning; HEW; Health extension worker; SNNPR: South Nation Nationality People Region; TV: Television

Knowledge about SRH services and HEP related perception

Only one hundred and seventeen (12.97%) young girls knew at least one method of FP and 97(10.75%) young girls were knowledgeable about modes of HIV transmission and prevention. Based on answers of strongly agree or moderately agree to attitude-related questions, six hundred and fifty-one (76.77%) young girls had a good perception of the HEW services being friendly to young's FP needs, 602(71.92) self-reported good trust in HEW and 732 (86.73%) had a good perception of HEP services. Three hundred forty-nine (38.69%) young girls recommended an increase in the frequency of home visits by HEWs (Table 2).

Table 2: Knowledge and perception of young girls on sexual and reproductive health-related health extension package in Ethiopia, 2020.

Variables	Number	Percent
Knowledge of FP methods		
Knowledgeable	117	12.97
Not knowledgeable	785	87.03
Comprehensive knowledge about HIV		
Knowledgeable	97	10.75
Not knowledgeable	805	89.25
Have a good perception of the HEW code of conduct		
Strongly disagree	41	4.83
Moderately disagree	156	18.40
Moderately agree	490	57.78
Strongly agree	161	18.99

For peer review only - http://bmjopen.bmj.com/site/about/guidelines.xhtml

Have good trust in HEW		
Strongly disagree	36	4.30
Moderately disagree	199	23.78
Moderately agree	438	52.33
Strongly agree	164	19.59
Have good perception on SRH service delivery by HEWs		
Strongly disagree	31	3.67
Moderately disagree	81	9.60
Moderately agree	442	52.37
Strongly agree	290	34.36
Which SRH related HEP services do you know?		
Health education	633	71.08
Family planning	540	60.39
Antenatal care	534	58.82
Delivery service	269	30.49
Postnatal care	359	40.39
Allocation of time for HP and home visit		
Increase the frequency of home visit	349	38.69
Increasing the time, they spend at HP	131	14.52
Increase both of them	347	38.47
Keep the current allocation of time	75	8.31

Utilization of SRH services

National level SRH services utilization of young girls was 19.18% (95% CI [16.74, 21.89]. Among the 244 young girls that live in pastoralist areas only 29 (11.90%) utilized SRH services. Similarly, among 658 young girls that live in agrarian areas, 144(21.9%) utilized SRH services. There is a significant amount of variation in the status of SRH utilization across the region accounting 17.16% of the total variability [(ICC=17.16 % ; 95% CI (6.30, 39.99)].

SRH services use among young girls were significantly higher for girls who had exposure to HEP through HEWs' home visits, HEWs' school visits, or during HP visits (70.5%) as compared to girls who did not have such exposure (37.6%).

Determinants of SRH services utilization use among young girls

Young girls who were exposed to HEW were three times more likely to utilize SRH services than girls who were not exposed to HEW [AOR=3.13, 95% CI, (2.03, 4.85)].

For peer review only

For peer review only - http://bmjopen.bmj.com/site/about/guidelines.xhtml

Other factors that are associated with increased SRH services utilization are knowing SRH related services are available at HPs [aOR=3.05, 95%CI (1.75, 5.33)], having good trust in HEW [aOR=1.82, CI (1.07, 3.10)], being in the age range of 20-24 years [aOR=2.93, CI (1.86, 4.63)], and being ever married [aOR=6.54, CI (3.82, 11.19)] compared to their counterparts (Table 3).

Table 3: Individual and region level factors associated with young girls' SRH service utilization from HEP in Ethiopia, 2020.

Variables	Utilization of SRH		COR (95% CI)	AOR (95% CI)
	No	Yes		
Exposure to HEW				
Not exposed	62.41	29.48	1.00	1.00
Exposed	37.59	70.52	3.61(2.47, 5.26)	3.13(2.03, 4.85)
Understanding HEP service availability				
Inadequate	91.50	8.50	1.00	1.00
Adequate	75.66	24.34	3.53 (2.19, 5.69)	3.05 (1.75, 5.33)
Trust in HEW				
Good	87.66	12.34	2.17(1.38, 3.42)	1.82(1.07, 3.10)
Poor	77.08	22.92	1.00	1.00
Age				
15-19	86.13	13.87	1.00	1.00
20-24	59.67	40.33	4.44 (3.01, 6.55)	2.93 (1.86, 4.63)
Marital status				
Ever Married	86.39	13.61	8.24 (5.17, 13.13)	6.54 (3.82, 11.19)
Never married	45.53	54.47	1.00	1.00

Note: HEW: Health Extension Worker, HEP: Health Extension Program, SRH: Sexual and Reproductive Health

Discussion

In this study, the status of SRH service utilization of young girls was very however, it was increased when they were exposed to HEP through health education provided by HEW. Exposure to HEWs, knowing about HEP service availability, and having trust in HEW were significantly associated with increased SRH service utilization among young girls. While older age and being ever married also were significantly associated with increased SRH service

For peer review only - http://bmjopen.bmj.com/site/about/guidelines.xhtml

utilization, educational status of young girls, wealth index of the household, perception about SRH service delivery by HEW, and perception of the youth-friendliness of HEWs were not significantly associated with SRH service utilization of young girls.

SRH service utilization of young girls was relatively lower compared to the findings of previous studies in Ethiopia (20–23). One possible reason for the discrepancy might be the difference in the study settings; participants for the current study are from rural areas where resources and access to services are very limited (23). Another study also explained that, this low utilization might also be linked to a low level of knowledge among young girls implying that focus given to young girls in rural areas by HEWs is likely to be minimal (24). This assumption is also supported by the low percentage of young girls in the current study having exposure to HEP services during a home visit or during their schooling. Encouraging HEWs to give due attention to young girls in rural areas might be one of the strategies that should be followed to increase these girls' service uptake in rural Ethiopia, which is the home of the significant majority of the population, which implies that investing out there can improve the country in general (13).

One of the HEP packages was designed to increase youth SRH service utilization through health education during a home visit, through school visits, and during HP-based service provision (24,25). In line with this, the current study findings showed that HEWs had a significant role in increasing the SRH service utilization of young girls. Based on the 2015 Ethiopian second generation HEP plan, whenever the HEWs meet young girls in their home, at school, or at HP, the odds of young girl's SRH service utilization was increased(13). This is a general indication that strengthening the role of the HEWs in implementing HEP strategies can improve SRH service utilization of young girls.

An adequate knowledge of available components of HEP is also significantly associated with increased service utilization among young girls. Among different components of SRH related HEP packages, most of the respondents recalled health education and FP services. This may reflect the differential focus given to FP education by HEWs during service delivery. Studies in Ethiopia and Uganda showed that knowing the place of SRH service increases service utilization(25,26). One of the global quality standard measures also indicates that someone is knowledgeable about their health only if they know where and when to get those services (27).

For peer review only - http://bmjopen.bmj.com/site/about/guidelines.xhtml

This is based on the assumption that the more they know about the available services the more likely it is for them to utilize the service in the nearby HP.

Confidentiality is one of the major ethical codes in medicine and SRH service utilization require similar level of confidentiality because of its sensitivity (11,28). Young girls are very worried about confidentiality, and they do not want to be seen by their parents, friends, or other people they know (relatives, teachers, or neighbors) while receiving SRH services because this knowledge makes them vulnerable to stigma and mortification(29). The International Federation of Gynecology and Obstetrics (FIGO) recognizes that health professionals are obligated to adhere to their patients' confidentiality (30). In the current study young girls who have a good trust in HEWs were more likely to utilize SRH services than their counterparts. Other studies also reported that increasing the privacy and confidentiality of youths improved their service utilization (31,32). Young girls should be provided confidential and flexible service in youth a youth friendly manner which can increase their trust and service utilization(33,34).

In line with a previous Ethiopian study, conducted at Baher Dar, young girls at the age of 20-24 years were more likely to utilize SRH services than girls aged 15-19 years (32). Sexual and reproductive health demand increases with age due both to a change in marital status that could lead to an interest in birth spacing, and increased educational status that could increase their ability to understand the risk of exposure to sexual practice (8).

Although the findings of the Ethiopian Demographic Health Survey showed that many young girls are engaged in sexual intercourse before marriage, the issue of SRH is culturally sensitive and socially unacceptable for unmarried girls which may decrease service utilization among young girls (8). In agreement with this, unmarried young girls in the current study were less likely to utilize SRH services from HEP. The negative attitude of HEWs toward use of SHR services by unmarried young girls, which is mainly due to lack of training, was provided as a justification for similar findings in a previous Ethiopian study (35).

Some limitations of the current study include the survey's failure to assess the attitude of HEWs about SRH delivery to unmarried young girls which, as just discussed, likely makes young girls less likely to utilize SRH services from HEP. Other limitations include recall bias on the part of

For peer review only - http://bmjopen.bmj.com/site/about/guidelines.xhtml

299 young girls surveyed which might have underestimated the magnitude of SRH services utilized
300 by these girls. Further, the survey used both female and male data collectors; using male data
301 collectors for this kind of study may have affected the magnitude of SRH service utilization
302 reported. This study also did not ask about barriers that hinder HEWs from increasing youth
303 service utilization. Additionally, we didn't perform a qualitative study to assess the effect of
304 cultural barriers such as existing norms, beliefs, gender related barriers and etc. on SRH service
305 utilizing. However, using nationally representative data is one of the important strengths of the
306 current study.

307 **Conclusion**

308 This study demonstrates that SRH service utilization of young girls in rural Ethiopia is very low.
309 Our findings also indicate that HEWs have a significant contribution to improving SRH service
310 utilization of young girls through health education during home visits, during school visits, and
311 at HPs. However, more effort might be required to significantly increase the exposure of young
312 girls to HEP services to increase service uptake. Encouraging young girls to build trust in HEWs
313 and to make young girls know what SRH related services are available at the HP is the first step
314 to improve service uptake. HEWs need to give special focus on younger girls and never married
315 young girls to increase their service uptake.

316 **Acknowledgment**

317 We are very thankful for MERQ consultancy for granting us to access this data source for this
318 study. We acknowledge Ms. Saron who thoroughly edited this manuscript for language usage,
319 spelling and grammar. Pre-Publication Support Service (PREPSS) supported the development of
320 this manuscript by providing pre-publication peer-review and copy editing.

321 **Competing interests:** The authors have declared that they do not have any competing interests
322 exist.

323

324 **Funding:** The study was conducted by MERQ Consultancy PLC as part of a national assessment
325 on the Health Extension Program of Ethiopia. The national assessment was financed through a
326 grant from the Bill & Melinda Gates Foundation (INV010174). The funder provided financial

For peer review only

For peer review only - http://bmjopen.bmj.com/site/about/guidelines.xhtml

support to MERQ that covered professional fees for leaders but did not have any additional role in the study design, data collection and analysis, decision to publish, or preparation of the manuscript. The specific roles of these authors are articulated in the “author contributions” section.

Author Contributions: AMT YK GM TLD contributed to the conceptualization, designing and conduct of the national HEP assessment, MGJ TLD MJ YS conceptualized the idea coved within this paper, MJ drafted the MS, MB lead the data analysis and involved in drafting of the MS. GM MGJ TLD supervised the write-up of the draft, all coauthors critically reviewed the drat MS and contributed intellectual inputs to improve the content of the MS and endorsed the final version of the MS to be submitted for possible publication.

Data availability: questionnaire and STATA do file

References

1. UN. Youth and the 2030 agenda for sustainable development. 2019. Available from: https://www.un.org/development/desa/youth/wp-content/uploads/sites/21/2018/12/WorldYouthReport-2030Agenda.pdf

2. United Nations. World Population Monitoring Adolescent and Youth Department of Economic and Social Affairs Population Division [Internet]. United Nations. 2012. Available from: http://www.un.org/en/development/desa/population/publications/pdf/fertility/12_66976_adolescents_and_youth.pdf

3. FMOH. Adolescence And Youth Health(AYH) Case Team | FMOH [Internet]. 2016 [cited 2021 Mar 23]. Available from: http://www.moh.gov.et/index.php/am/AYH

4. United Nations. World population prospects 2019. Department of Economic and Social Affairs. World Population Prospects 2019. 2019.

5. UNFPA. The Case for Investing in Young People as part of a National Poverty Reduction Strategy [Internet]. 2005. Available from: http://www.unfpa.org/webdav/site/global/shared/documents/publications/2005/case_youn

For peer review only

For peer review only - http://bmjopen.bmj.com/site/about/guidelines.xhtml

gpeople_eng.pdf

6. WHO. World health statistics overview:monitoring health for the SDGs. 2019. Available from: https://apps.who.int/iris/handle/10665/324835.

7. Dehne KL, Riedner G. Sexually transmitted infections among adolescents: The need for adequate health services. Reprod Health Matters. 2001;9(17):170–83. doi: 10.1016/s0968-8080(01)90021-7. PMID: 11468834.

8. Central Statistical Agency [Ethiopia], ICF International. Ethiopia Demographic and Health Survey 2011. Ethiop Water Sect Dev Progr [Internet]. 2012;1–452. Available from: https://www.usaid.gov/sites/default/files/documents/1860/Ethiopia DHS 2016 KIR - Final 10-17-2016.pdf

9. Amare T, Yeneabat T, Amare Y. A Systematic Review and Meta-Analysis of Epidemiology of Risky Sexual Behaviors in College and University Students in Ethiopia , 2018. *J Environ Public Health*. 2019 Mar 20;2019:4852130. doi: 10.1155/2019/4852130. PMID: 3101584

10. Ethiopian Public Health Institute (EPHI) [Ethiopia] and ICF. Ethiopia Mini 396 Demographic and Health Survey 2019: Final Report. Rockville, Maryland, USA: EPHI and 397 ICF 2021. Available from: https://www.unicef.org/ethiopia/media/1721/file/The%202019%20Ethiopia%20Mini%20Demographic%20and%20Health%20Survey%20.pdf

11. Birhan Z, Tushune K, Jebena MG. Sexual and reproductive health services use, perceptions, and barriers among young people in southwest Oromia, Ethiopia. *Ethiopian journal of health sciences.* 2018 Jan 10;28(1):37-48.

12. kidanu, kiros G. Health Sector Development Program Four(HSDP-IV) Available from http://tucghe.org/HSDP%20IV.pdf

13. FMOH. Ethiopia second Generation Health Extension Program. 2015. Available from: https://publications.jsi.com/JSIInternet/Inc/Common/_download_pub.cfm?id=16833&lid=3

For peer review only

For peer review only - http://bmjopen.bmj.com/site/about/guidelines.xhtml

14. Alebachew Abebe, Waddington & Catriona.. Improving health system efficiency: Ethiopia human resources for health reforms. 2015. Available from: https://apps.who.int/iris/handle/10665/187240.

15. Kemal, Mohammed. Federal Democratic Republic of Ethiopia Ministry of Health Adolescent Reproductive Health Extension Package. Available from: https://www.researchgate.net/publication/332410714_Federal_Democratic_Republic_of_Ethiopia_Ministry_of_Health_Adolescent_Reproductive_Health_Extension_Package

16. Federal democratic republic of Ethiopia Ministry of Health, Health sector transformation Plan (HSTP). 2015. Available from: https://www.globalfinancingfacility.org/sites/gff_new/files/Ethiopia-health-system-transformation-plan.pdf

17. Merq conseltancy PPLC. HEP [National Assessment of The Ethiopian Health Extension Program] REPORT - MERQ Consultancy. 2021 [cited 2021 Nov 18]. p. 1–43. Available from: https://merqconsultancy.org/p/22/hep-national-assessment-of-the-ethiopian-health-extension-program-report

18. Yitayal M., Et.al. Health extension program factors, frequency of household visits and being model households, improved utilization of basic health services in Ethiopia. *BMC Health Serv Res*. 2014;14:156. Available from: http://ovidsp.ovid.com/ovidweb.cgi?T=JS&PAGE=reference&D=emed12&NEWS=N&AN=24708661

19. Ethiopia Central Statistical Agency (CSA), Ethiopia Demographic and Health Survey 2016, Central Statistical Agency, Addis Ababa & ICF International, Calverton, MD, USA, 2016.Available from: https://dhsprogram.com/pubs/pdf/FR328/FR328.pdf

20. Amanuel AA, Seme AS. Reproductive Health Knowledge and Services Utilization among Rural Adolescents in Machakal district, Northwest Ethiopia. *Asian Journal of Pharmacy*, Nursing and Medical Sciences. 2013 May 25;1(1).

21. Binu W, Marama T, Gerbaba M, Sinaga M. Sexual and reproductive health services

For peer review only

For peer review only - http://bmjopen.bmj.com/site/about/guidelines.xhtml

utilization and associated factors among secondary school students in Nekemte town, Ethiopia. *Reprod Health.* 2018;Dec15(1):1–0. 22.

22. Gebreyesus H, Teweldemedhin M, Mamo A. Determinants of reproductive health services utilization among rural female adolescents in Asgede-Tsimbla district Northern Ethiopia: A community based cross-sectional study. *Reprod Health*. 2019;Dec16(1):1–10.

23. Ayehu A, Kassaw T HG. Level of young people sexual and reproductive health service utilization and its associated factors among young people in Awabel District, Northwest Ethiopia. *PLoS One*. 2016;Mar 18; 11(3): e0151613

24. Yitayal M, Berhane Y, Worku A, et.al. Health extension program factors , frequency of household visits and being model households , improved utilization of basic health services in Ethiopia. *BMC Health services research* 2014 Dec;14(1):1-9. doi: 10.1186/1472-6963-14-156

25. Na M, Jennings L, Talegawkar SA, Ahmed S. Association between women's empowerment and infant and child feeding practices in sub-Saharan Africa: an analysis of Demographic and Health Surveys. *Public health nutrition*. 2015. Dec;18(17): 3155–65.

26. Nair M, Baltag V, Bose K, Boschi-Pinto C, Lambrechts T, Mathai M. Improving the Quality of Health Care Services for adolescents , globally : a standards-driven approach. *Journal of Adolescent Health*. 2015 Sep 1;57(3):288-98.

27. FIGO. International Federation of Gynecology and Obstetrics. Ethical Issues in Obstetrics and Gynecology. FIGO Committee for the Study of Ethical Aspects of Human Reproduction and Women's Health. London. 2012. 1–148 p.

28. ACHE Code of Ethics | American College of Healthcare Executives [Internet]. [cited 2021 Mar 24]. Available from: https://www.ache.org/about-ache/our-story/our-commitments/ethics/ache-code-of-ethics

29. Adolescent and Youth-Friendly Services | The Challenge Initiative [Internet]. [cited 2021 Jul 3]. Available from: https://tciurbanhealth.org/courses/adolescent-youth-sexual-reproductive-health-services-supply/lessons/adolescent-and-youth-friendly-services-3/

For peer review only

30. Denno DM, Hoopes AJ, Chandra-Mouli V. Effective strategies to provide adolescent sexual and reproductive health services and to increase demand and community support. *J Adolesc Health*. 2015;56(1):S22–41. Available from: http://dx.doi.org/10.1016/j.jadohealth.2014.09.012

31. Feleke SA, Koye DN, Demssie AF, Mengesha ZB. Reproductive health service utilization and associated factors among adolescents (15-19 years old) in Gondar town, Northwest Ethiopia. *BMC Health services resarch*. 2013;Dec;13(1):1–0.

32. Abebe M, Awoke W. Utilization of Youth Reproductive Health Services and Associated Factors among High School Students in Bahir Dar, Amhara Regional State, Ethiopia. *Open Journal of Epidemiology*. 2014;Mar27(02):69–75.

33. Haile B, Shegaze M, Feleke T, Glagn M, et.al. Disparities in utilization of sexual and reproductive health services among high school adolescents from youth friendly service implemented and non-implemented areas of Southern Ethiopia. *Arch Public Heal*. 2020 Dec 1 [cited 2021 Mar 23];78(1):126. Available from: https://archpublichealth.biomedcentral.com/articles/10.1186/s13690-020-00508-w

34. Amsale C, Yemane B. Knowledge of sexually transmitted infections and barriers to seeking health services among high school adolescents in Addis Ababa, Ethiopia. *Journal of AIDS and Clinical Research*. 2012;3(5).35.

35. Tilahun M, Mengistie B, Egata G, Reda AA. Health workers ’ attitudes toward sexual and reproductive health services for unmarried adolescents in Ethiopia. *Reproductive health*. 2012;Dec;9(1):1–7, doi: https://doi.org/10.1186/1742-4755-9-19.

For peer review only - http://bmjopen.bmj.com/site/about/guidelines.xhtml

STROBE Statement—Checklist of items that should be included in reports of ***cross-sectional studies***

	Item No	Recommendation	Page No
**Title and abstract**	1	(*a*) Indicate the study's design with a commonly used term in the title or the abstract	1 - 2
		(*b*) Provide in the abstract an informative and balanced summary of what was done and what was found	1 - 2
**Introduction**			
Background/rationale	2	Explain the scientific background and rationale for the investigation being reported	3
Objectives	3	State specific objectives, including any prespecified hypotheses	4
**Methods**			
Study design	4	Present key elements of study design early in the paper	5
Setting	5	Describe the setting, locations, and relevant dates, including periods of recruitment, exposure, follow-up, and data collection	4-5
Participants	6	(*a*) Give the eligibility criteria, and the sources and methods of selection of participants	5
Variables	7	Clearly define all outcomes, exposures, predictors, potential confounders, and effect modifiers. Give diagnostic criteria, if applicable	6
Data sources/ measurement	8*	For each variable of interest, give sources of data and details of methods of assessment (measurement). Describe comparability of assessment methods if there is more than one group	5
Bias	9	Describe any efforts to address potential sources of bias	5-6
Study size	10	Explain how the study size was arrived at	5
Quantitative variables	11	Explain how quantitative variables were handled in the analyses. If applicable, describe which groupings were chosen and why	7
Statistical methods	12	(*a*) Describe all statistical methods, including those used to control for confounding	7
		(*b*) Describe any methods used to examine subgroups and interactions	7
		(*c*) Explain how missing data were addressed	7
		(*d*) If applicable, describe analytical methods taking account of sampling strategy	7
		(*e*) Describe any sensitivity analyses	7
**Results**			
Participants	13*	(a) Report numbers of individuals at each stage of study—eg numbers potentially eligible, examined for eligibility, confirmed eligible, included in the study, completing follow-up, and analysed	8
		(b) Give reasons for non-participation at each stage	8
		(c) Consider use of a flow diagram	8
Descriptive data	14*	(a) Give characteristics of study participants (eg demographic, clinical, social) and information on exposures and potential confounders	8
		(b) Indicate number of participants with missing data for each variable of interest	8
Outcome data	15*	Report numbers of outcome events or summary measures	10
Main results	16	(*a*) Give unadjusted estimates and, if applicable, confounder-adjusted estimates and their precision (eg, 95% confidence interval). Make clear which confounders were adjusted for and why they were included	10

For peer review only

For peer review only - http://bmjopen.bmj.com/site/about/guidelines.xhtml

		(*b*) Report category boundaries when continuous variables were categorized	10
		(*c*) If relevant, consider translating estimates of relative risk into absolute risk for a meaningful time period	11
Other analyses	17	Report other analyses done—eg analyses of subgroups and interactions, and sensitivity analyses	11
**Discussion**			
Key results	18	Summarise key results with reference to study objectives	11
Limitations	19	Discuss limitations of the study, taking into account sources of potential bias or imprecision. Discuss both direction and magnitude of any potential bias	13
Interpretation	20	Give a cautious overall interpretation of results considering objectives, limitations, multiplicity of analyses, results from similar studies, and other relevant evidence	11-13
Generalisability	21	Discuss the generalisability (external validity) of the study results	14
**Other information**			
Funding	22	Give the source of funding and the role of the funders for the present study and, if applicable, for the original study on which the present article is based	

*Give information separately for exposed and unexposed groups.

Note: An Explanation and Elaboration article discusses each checklist item and gives methodological background and published examples of transparent reporting. The STROBE checklist is best used in conjunction with this article (freely available on the Web sites of PLoS Medicine at http://www.plosmedicine.org/, Annals of Internal Medicine at http://www.annals.org/, and Epidemiology at http://www.epidem.com/). Information on the STROBE Initiative is available at www.strobe-statement.org.

For peer review only

BMJ Open

Sexual and reproductive health service utilization of young girls in rural Ethiopia: What are the roles of health extension workers?: Community based cross-sectional study

Journal:	*BMJ Open*
Manuscript ID	bmjopen-2021-056639.R2
Article Type:	Original research
Date Submitted by the Author:	23-Aug-2022
Complete List of Authors:	Jisso, Meskerem ; Hawassa University, Public health<br>Feyasa, Merga; Addis Ababa University College of Natural Sciences, Statistics<br>Medhin, Girmay; Addis Ababa University, Aklilu Lemma Institute of Pathology<br>Dadi, Tegene ; Hawassa University College of Medicine and Health Sciences, School of Public health<br>Simachew, Yilkal; Hawassa University College of Medicine and Health Sciences, School of public health<br>Denberu, Bisrat; federal ministry of health<br>Jebena, Mulusew; Jimma University College of Public Health and Medical Sciences<br>Alemayehun, Yibeltal ; MERQ Consultancy PLC<br>Teklu, Alula M.; MERQ Consultancy PLC ; MERQ PLC LLC, research
**Primary Subject Heading**:	Sexual health
Secondary Subject Heading:	Sexual health, Public health
Keywords:	REPRODUCTIVE MEDICINE, PUBLIC HEALTH, SEXUAL MEDICINE, EPIDEMIOLOGY, EDUCATION & TRAINING (see Medical Education & Training), STATISTICS & RESEARCH METHODS

SCHOLARONE™
Manuscripts

I, the Submitting Author has the right to grant and does grant on behalf of all authors of the Work (as defined in the below author licence), an exclusive licence and/or a non-exclusive licence for contributions from authors who are: i) UK Crown employees; ii) where BMJ has agreed a CC-BY licence shall apply, and/or iii) in accordance with the terms applicable for US Federal Government officers or employees acting as part of their official duties; on a worldwide, perpetual, irrevocable, royalty-free basis to BMJ Publishing Group Ltd ("BMJ") its licensees and where the relevant Journal is co-owned by BMJ to the co-owners of the Journal, to publish the Work in this journal and any other BMJ products and to exploit all rights, as set out in our licence.

The Submitting Author accepts and understands that any supply made under these terms is made by BMJ to the Submitting Author unless you are acting as an employee on behalf of your employer or a postgraduate student of an affiliated institution which is paying any applicable article publishing charge ("APC") for Open Access articles. Where the Submitting Author wishes to make the Work available on an Open Access basis (and intends to pay the relevant APC), the terms of reuse of such Open Access shall be governed by a Creative Commons licence – details of these licences and which Creative Commons licence will apply to this Work are set out in our licence referred to above.

Other than as permitted in any relevant BMJ Author's Self Archiving Policies, I confirm this Work has not been accepted for publication elsewhere, is not being considered for publication elsewhere and does not duplicate material already published. I confirm all authors consent to publication of this Work and authorise the granting of this licence.

For peer review only

Sexual and reproductive health service utilization of young girls in rural Ethiopia: What are the roles of health extension workers? : Community based cross-sectional study

Meskerem Jisso[1]*, Merga Belina Feyasa[2], Girmay Medhin[3,4], Tegene Legese Dadi[1], Yilkal Simachew[1], Bisrat Denberu[5], Mulusew Gerbaba Jebena [6], Yibeltal Kifle Alemayehu[6,4] Alula Meresa Teklu[4]

Affiliation

1: Hawassa University College of medicine and health science, School of Public Health

2: Addis Ababa University, Department of Statistics

3: Addis Ababa University, Aklilu Lemma Institute of Pathobiology

4: MERQ consultancy PLC

5: Federal minister of health, Ethiopia

6: Jimma University Institute of Health

***Corresponding author**

Meskerem Jisso

1: Hawassa University College of medicine and health science, School of public health

Email: Meskyj@gmail.com

For peer review only

For peer review only - http://bmjopen.bmj.com/site/about/guidelines.xhtml

Abstract

Objective: Little is known about the extent to which Health Extension Program (HEP) has played its role to increase service uptake among young girls. This study aims to estimate the status of young girls' SRH services utilization in rural Ethiopia and to examine the role of health extension workers (HEWs) in this regard.

Design: A cross-sectional study.

Setting: A community-based study among all nine regions of Ethiopia.

Participants: nine hundred and two young girls aged 15-24 years were included in this study.

Method: We used data from the national HEP assessment, collected from March to May 2019. Multilevel binary logistic regression was used to investigate the association between exposure to HEP and sexual and reproductive health (SRH) services utilization of young girls and we reported an adjusted odds ratio with a corresponding 95% confidence interval (CI) as measure of the degree of associations.

Result: Only 19.18% [95% CI (16.74, 21.89)] of young girls used SRH services with significant regional variability (Intra-regional variability [(ICC) =17.16 %; 95% CI (6.30, 39.99)]. Exposure to HEP [aOR=3.13, 95%CI, (2.03, 4.85)], knowing about the availability of HEP services [aOR=3.06, 95%CI (1.75, 5.33)], and having good trust in HEWs [AOR=1.82, CI (1.07, 3.10)], and other sociodemographic factors were significantly associated with increased SRH services utilization.

Outcome: SRH service utilization

Conclusion: Although the overall SRH service utilization of young girls in rural Ethiopia was very low, HEWs have a great contribution to improving service utilization of young girls through strong health education provided during home visits, school visits, and at Health Posts. More investment along this line has the potential to improve service uptake among young girls. Encouraging HEWs to build trust among this segment of the population and creating awareness of SRH related services is crucial to improv service uptake.

For peer review only - http://bmjopen.bmj.com/site/about/guidelines.xhtml

Strength and limitation of this study

- The study was done at country level, which is more representative.
- It did not assess the attitude of HEWs about SRH delivery
- Recall bias that may have underestimated the magnitude of SRH services.
- We could not assess the effect of cultural factors as a barrier for utilizing SRH service qualitatively.

Background

Youths between 15-24 years of age account for more than 1.2 billion people globally with 90% of them living in developing countries (1). In sub-Saharan Africa, this segment of the population constitutes 23% of the region's population (2). In Ethiopia, 34% of the total population is under 25 years and the majority of them (79% males and 78% females) reside in rural areas (3). The number of young people in the least developed countries is expected to rise from 207 million in 2019 to 336 million by the year 2050 (4).

Having healthy youth leads to improved outcomes for a family, society, and a nation as a whole. Hence, investing in their health has the potential to break the cycle of poverty through increased productivity, decreased health costs, and improved social capital (5). Yet young girls are undergoing great physical, physiological and emotional changes, which aggravate their vulnerability to sexual and reproductive health (SRH) problems (5). Globally, in 2018, one in 25 young girls was at risk of early pregnancy, which is the leading cause of mortality among young girls (6). Globally, three million young girls undergo an unsafe abortion, 460,000 were newly infected with HIV and 333 million of them were infected with a sexually transmitted infection (STI) (7). Similarly, in Ethiopia by 2016, 0.4% of youths were infected by HIV/AIDS (8), and among university and college students, based on a 2019 study, 41.62% engaged in risky sexual behavior (9).

Previous studies in Ethiopia showed that the SRH knowledge of young girls and their SRH service utilization is very low, which might worsen their SRH problems (3). A survey conducted in 2019 showed that only 37% of married young girls utilized family planning (FP) services (10).

For peer review only - http://bmjopen.bmj.com/site/about/guidelines.xhtml

On the other hand, one-third of married and 12% of unmarried young girls have an unmet need for FP (10). Lack of SRH related information, poor perceptions about SRH, feelings of shame, fear of being seen by others, while they are utilizing the SRH services, restrictive cultural norms and inaccessibility to health services are among the underlying causes for this low service utilization (3,11).

Cognizant of these facts, the Ethiopian government launched a health extension program (HEP) in 2003 as a community-based program to increase the quality and access to health services in order to prevent disease, promote healthy behaviors, and improve knowledge and service utilization (12,13). Youth reproductive health is one of the 18 HEP packages designed to be delivered at a grass-root level (13). HEWs are expected to educate the community on these packages, and specifically on SRH related issues, and provide different health services through a home visits, school visits, and at health posts (HPs) (14). Nonetheless, to date, to the best of our knowledge, there is limited evidence about the extent to which SRH services are utilized by young girls (15). Having such evidence is useful to monitor the performance of HEP in addressing one of the health service uptakes by young girls. This study aims to assess the level of SRH service utilization of young girls in rural Ethiopia and to examine the role of HEP in this regard.

Methods

Study Context

During the data collection Ethiopia was administratively divided into nine regions and two city administrations. Each region is hierarchically divided into zones, districts, and then kebeles. A kebele is the smallest administrative unit expected to serve an average of 5,000 people, and a district is the lowest budgeted center of the government. More than 95% of the kebeles in the country have HPs. HPs are categorized under the primary level of Ethiopian health system tiers, constructed within each kebele and staffed on average by two HEWs. Each HEW is expected to serve 1,500 up to 2,500 people and there are nearly to 40,000 HEWs in more than 17,000 HPs in Ethiopia.

For peer review only

HEWs are high school graduates with an additional one year of education that enables them to be engaged in health education and delivery of FP methods and selected curative services. They are expected to spend 75% of their time in outreach services (home visits, school visits, and community campaigns) and 25% on curative services at the HP. For the success of the HEP program, HEWs are supervised at least weekly and monitored by the nearby primary health care unit health professionals (16). Health education on SRH issues is supposed to be provided by HEWs both at health posts and at the household level. Services like Skilled birth, selected FP methods (intrauterine devices, Norplant, and permanent FP), and treatment for STI or HIV are provided at the health center or hospital level rather than at the HP level. In these cases, HEWs are expected to refer the clients to the catchment health center after counseling them (13).

Study design, setting and data source

A cross-sectional national survey was conducted using a multistage sampling method to evaluate the Ethiopian rural HEP, and for this study, data were extracted from this survey. The survey covered all the nine administrative regions of the country and data collection was conducted from March to May 2019. In total, 62 (both agrarian and pastoralist regions) districts were covered in the assessment by using appropriate methodological flow. Within each district three kebeles were randomly selected and then within each selected kebeles, 34 households were randomly selected from the total list of households in the kebele. A total of 902 young girls between the age of 15-24 years from a total of 6,324 rural households were included in the HEP program evaluation assessment and we have used these data for analysis. The detailed methods and sampling procedures are described elsewhere (17).

Data collection for the HEP assessment

During the HEP program evaluation assessment, we used a structured questionnaire adapted from different literature (18–20). Interview administered field data collection was facilitated using Open Data Kit (ODK) application with young girls at each kebele. The questionnaire was translated into different local languages of Ethiopia and all study participants were interviewed in their local languages. Before starting the actual data collection, the questionnaire was pre-tested for all languages out of the actual study area then any language barriers and jargon words were corrected and missed valuable variables were also added to the questionnaire. To minimize

For peer review only - http://bmjopen.bmj.com/site/about/guidelines.xhtml

recall bias different techniques such as probing questions, and decreasing the study period were used. Data collectors (both male and female) were recruited and given 10-day training on study objectives, data collection methods, ethical procedures, and data quality issues.

Patient and Public Involvement

No patients involved

Measurements

Outcome variable

Young girls' sexual and reproductive health service utilization: This is a dichotomous outcome variable that refers to the status of a young girl in utilizing at least one of SRH services: (a) attending health education on SRH issue (during a home visit or at school or during HP visit) or (b) FP utilization (utilization of at least one modern FP method like IUCD, implants, injectable, condoms, pills). The variable assumes a value of "1" if a given young girl utilized at least one of the SRH services and "0" if she did not utilize any.

Key Exposure variable

Exposure to HEW: This is the exposure variable that we used to investigate the role of HEP in enhancing SRH service utilization of young girls. We measured this variable as follows: young girls who meet HEW for any service at a home visit, school visit, or HP visits within a one-year period prior to data collection time were labeled as "exposed to HEW" or else they were labeled as "Not exposed".

Another independent variable

Socio-demographic characteristics: age, educational status, marital status (categorized as never married and ever married (married, separated, and widowed)), source of information for FP, region, and wealth index of the household (categorized as lower, middle, and high).

Trust in HEW: This is a composite variable constructed from seven perception-related questions, intended to measure a young woman's attitude toward HEWs. Those young girls who answered strongly agree or moderately agree to all seven perception-related questions were

For peer review only - http://bmjopen.bmj.com/site/about/guidelines.xhtml

considered to have "good trust in HEWs" and those who answered strongly disagree or moderately disagree were labeled as "poor trust in HEWs".

Knowing the availability of SRH-related HEP services at HP : Young girls who know at least two SRH related HEP packages/services (health education, delivery, antenatal care (ANC), postnatal care (PNC), FP, HIV test) were considered as having an "adequate knowledge" and the other young girls were considered as having "inadequate knowledge".

Modern FP method knowledge: young girls who know at least one modern FP method (i.e. injectable, pills, Intrauterine device (IUD), condom (both male and female) and implants) were labeled as "knowledgeable about modern FP methods", and those who were not able to list any one of the five methods were considered as "not knowledgeable about modern FP methods".

Knowledge of HIV transmission: This is another composite variable that used responses from nine questions. A young girl who gave at least one correct answer from a total of nine HIV transmission-related questions was labeled as having "knowledge of HIV transmission" and if she did not give a correct answer for any of the nine HIV transmission-related questions, she was considered as "not knowledgeable about HIV transmission"

Statistical Analysis

We used Stata 14 for the analysis and reported frequencies, percentages, mean, and standard deviations as descriptive summary measures of key variables. We used multilevel binary logistic regression to model the odds of utilizing SRH services. Due to the multistage cluster sampling procedure, individual young girls were nested within kebeles and then within regions. We examined the effect of the individual-level variables, and the region-level variables using two-level logistic regression modeling. During analysis, the characteristics of young girls were taken as individual level (level-1), and regions were taken as level-2. The region was considered as level two to understand regional variability which is useful for future intervention.

Based on this model, the intra-class correlation coefficient (ICC) was calculated to evaluate whether the variation in the odds of utilizing SRH services was primarily within or between regions. Both the crude odds ratio (cOR) and the adjusted odds ratio (aOR) with their

For peer review only - http://bmjopen.bmj.com/site/about/guidelines.xhtml

corresponding 95% Confidence Interval (CI) were reported as measures of strength of association.

Ethical consideration

The ethical letter of approval was obtained from ETHIOPIAN PUBLIC HEALTH ASSOCIATION (1613/820) and informed consent was obtained from the participants.

Results

Socio-demographic characteristics

A total of 902 young girls were interviewed, yielding a 100% response rate. The mean age of the respondents was 17.48 years (SD = ±2.27), 816 (80%) young girls were in the age range of 15-19 years, 170 (18.82%) had no formal education, and 779 (86.36%) were never married (Table 1).

Table 1: socio-demographic and household-level characteristics of young girls in Ethiopia, 2020.

Characteristics	Number	Percent
Age		
15-19	721	79.90
20-24	181	20.10
Educational status		
No formal education	170	18.85
Primary	514	56.98
Secondary and above	218	24.17
Marital Status		
Single	779	86.36
Ever married	123	13.64
Region		
Tigray	117	12.97
Afar	63	6.98
Amhara	169	18.74
Oromia	162	17.96
Somali	97	10.75
Benishangul Gumuz	50	5.54
SNNPR	150	16.63
Gambela	36	3.99
Harari	58	6.43
Source of information for FP		
HEW	186	23.4

For peer review only - http://bmjopen.bmj.com/site/about/guidelines.xhtml

Other health professionals	52	6.54
Other sources (TV, radio, friend, relatives, and printed materials)	557	70.06
Wealth index		
Lower	290	32.88
Middle	186	21.09
Higher	406	46.03

Note: FP: Family Planning; HEW; Health extension worker; SNNPR: South Nation Nationality People Region; TV: Television

Knowledge about SRH services and HEP related perception

Only one hundred and seventeen (12.97%) young girls knew at least one method of FP and 97(10.75%) young girls were knowledgeable about modes of HIV transmission and prevention. Based on answers of strongly agree or moderately agree to attitude-related questions, six hundred and fifty-one (76.77%) young girls had a good perception of the HEW services being friendly to young's FP needs, 602(71.92) self-reported good trust in HEW and 732 (86.73%) had a good perception of HEP services. Three hundred forty-nine (38.69%) young girls recommended an increase in the frequency of home visits by HEWs (Table 2).

Table 2: Knowledge and perception of young girls on sexual and reproductive health-related health extension package in Ethiopia, 2020.

Variables	Number	Percent
Knowledge of FP methods		
Knowledgeable	117	12.97
Not knowledgeable	785	87.03
Comprehensive knowledge about HIV		
Knowledgeable	97	10.75
Not knowledgeable	805	89.25
Have a good perception of the HEW code of conduct		
Strongly disagree	41	4.83
Moderately disagree	156	18.40
Moderately agree	490	57.78
Strongly agree	161	18.99

For peer review only

For peer review only - http://bmjopen.bmj.com/site/about/guidelines.xhtml

Have good trust in HEW		
Strongly disagree	36	4.30
Moderately disagree	199	23.78
Moderately agree	438	52.33
Strongly agree	164	19.59
Good perception on SRH service delivery by HEWs		
Strongly disagree	31	3.67
Moderately disagree	81	9.60
Moderately agree	442	52.37
Strongly agree	290	34.36
Knowing the availability of SRH-related HEP services at HP		
Health education	633	71.08
Family planning	540	60.39
Antenatal care	534	58.82
Delivery service	269	30.49
Postnatal care	359	40.39
Allocation of time for HP and home visit		
Increase the frequency of home visits	349	38.69
Increasing the time, they spend at HP	131	14.52
Increase both of them	347	38.47
Keep the current allocation of time	75	8.31

Utilization of SRH services

National level SRH services utilization of young girls was 19.18% (95% CI [16.74, 21.89]. Among the 244 young girls that live in pastoralist areas, only 29 (11.90%) utilized SRH services. Similarly, among 658 young girls that live in agrarian areas, 144(21.9%) utilized SRH services. There is a significant amount of variation in the status of SRH utilization across the region accounting for 17.16% of the total variability [(ICC=17.16 %; 95% CI (6.30, 39.99)].

SRH services use among young girls was significantly higher for girls who had exposure to HEP through HEWs' home visits, HEWs' school visits, or during HP visits (70.5%) as compared to girls who did not have such exposure (37.6%).

Determinants of SRH services utilization use among young girls

For peer review only

For peer review only - http://bmjopen.bmj.com/site/about/guidelines.xhtml

Young girls who were exposed to HEW were three times more likely to utilize SRH services than girls who were not exposed to HEW [AOR=3.13, 95% CI, (2.03, 4.85)].

Other factors that are associated with increased SRH services utilization are knowing SRH-related services are available at HPs [aOR=3.05, 95%CI (1.75, 5.33)], having good trust in HEW [aOR=1.82, CI (1.07, 3.10)], being in the age range of 20-24 years [aOR=2.93, CI (1.86, 4.63)], and being ever married [aOR=6.54, CI (3.82, 11.19)] compared to their counterparts (Table 3).

For peer review only

Table 3: Individual and region level factors associated with young girls' SRH service utilization from HEP in Ethiopia, 2020.

Variables	Utilization of SRH		COR (95% CI)	AOR (95% CI)
	No	Yes		
Exposure to HEW				
Not exposed	62.41	29.48	1.00	1.00
Exposed	37.59	70.52	3.61(2.47, 5.26)	3.13(2.03, 4.85) *
Understanding HEP service availability				
Inadequate	91.50	8.50	1.00	1.00
Adequate	75.66	24.34	3.53 (2.19, 5.69)	3.05 (1.75, 5.33) *
Trust in HEW				
Good	87.66	12.34	2.17(1.38, 3.42)	1.82(1.07, 3.10) *
Poor	77.08	22.92	1.00	1.00
Age				
15-19	86.13	13.87	1.00	1.00
20-24	59.67	40.33	4.44 (3.01, 6.55)	2.93 (1.86, 4.63) *
Marital status				
Ever Married	86.39	13.61	8.24 (5.17, 13.13)	6.54 (3.82, 11.19) *
Never married	45.53	54.47	1.00	1.00

Note: HEW: Health Extension Worker, HEP: Health Extension Program, SRH: Sexual and Reproductive Health, *: P-value less than 0.05.

Discussion

In this study, the status of SRH service utilization of young girls was very low however, it was increased when they were exposed to HEP through health education provided by HEW.

For peer review only - http://bmjopen.bmj.com/site/about/guidelines.xhtml

Exposure to HEWs, knowing about HEP service availability, and having trust in HEW were significantly associated with increased SRH service utilization among young girls. While older age and being ever married also were significantly associated with increased SRH service utilization, educational status of young girls, wealth index of the household, perception about SRH service delivery by HEW, and perception of the youth-friendliness of HEWs were not significantly associated with SRH service utilization of young girls.

SRH service utilization of young girls was relatively lower compared to the findings of previous studies in Ethiopia (20–23). One possible reason for the discrepancy might be the difference in the study settings; participants in the current study are from rural areas where resources and access to services are very limited (23). Another study also explained that this low utilization might also be linked to a low level of knowledge among young girls implying that the focus given to young girls in rural areas by HEWs is likely to be minimal (24). This assumption is also supported by the low percentage of young girls in the current study having exposure to HEP services during a home visit or during their schooling. Encouraging HEWs to give due attention to young girls in rural areas might be one of the strategies that should be followed to increase these girls' service uptake in rural Ethiopia, which is the home of the significant majority of the population, which implies that investing out there can improve the country in general (13).

One of the HEP packages was designed to increase youth SRH service utilization through health education during home visits, school visits, and HP-based service provision (24,25). In line with this, the current study findings showed that HEWs had a significant role in increasing the SRH service utilization of young girls. Based on the 2015 Ethiopian second generation HEP plan, whenever the HEWs meet young girls in their home, at school, or HP, the odds of young girls' SRH service utilization were increased (13). This is a general indication that strengthening the role of the HEWs in implementing HEP strategies can improve the SRH service utilization of young girls.

Adequate knowledge of available components of HEP is also significantly associated with increased service utilization among young girls. Among different components of SRH related HEP packages, most of the respondents recalled health education and FP services. This may reflect the differential focus given to FP education by HEWs during service delivery. Studies in

For peer review only - http://bmjopen.bmj.com/site/about/guidelines.xhtml

Ethiopia and Uganda showed that knowing the place of SRH service increases service utilization (25,26). One of the global quality standard measures also indicates that someone is knowledgeable about their health only if they know where and when to get those services (27). This is based on the assumption that the more they know about the available services the more likely it is for them to utilize the service in the nearby HP.

Confidentiality is one of the major ethical codes in medicine and SRH service utilization requires a similar level of confidentiality because of its sensitivity (11,28). Young girls are very worried about confidentiality, and they do not want to be seen by their parents, friends, or other people they know (relatives, teachers, or neighbors) while receiving SRH services because this knowledge makes them vulnerable to stigma and mortification (29). The International Federation of Gynecology and Obstetrics (FIGO) recognizes that health professionals are obligated to adhere to their patients' confidentiality (30). In the current study young girls who have a good trust in HEWs were more likely to utilize SRH services than their counterparts. Other studies also reported that increasing the privacy and confidentiality of youths improved their service utilization (31,32). Young girls should be provided confidential and flexible service in a youth friendly manner which can increase their trust and service utilization (33,34).

In line with a previous Ethiopian study, conducted at Baher Dar, young girls between the age of 20-24 years were more likely to utilize SRH services than girls aged 15-19 years (32). Sexual and reproductive health demand increases with age due both to a change in marital status that could lead to an interest in birth spacing, and increased educational status that could increase their ability to understand the risk of exposure to sexual practice (8).

Although the findings of the Ethiopian Demographic Health Survey showed that many young girls are engaged in sexual intercourse before marriage, the issue of SRH is culturally sensitive and socially unacceptable for unmarried girls which may decrease service utilization among young girls (8). In agreement with this, unmarried young girls in the current study were less likely to utilize SRH services from HEP. The negative attitude of HEWs toward the use of SRH services by unmarried young girls, which is mainly due to lack of training, was provided as a justification for similar findings in a previous Ethiopian study (35).

For peer review only - http://bmjopen.bmj.com/site/about/guidelines.xhtml

Some limitations of the current study include the survey's failure to assess the attitude of HEWs about SRH delivery to unmarried young girls which, as just discussed, likely makes young girls less likely to utilize SRH services from HEP. Other limitations include recall bias on the part of young girls surveyed which might have underestimated the magnitude of SRH services utilized by these girls. Further, the survey used both female and male data collectors; using male data collectors for this kind of study may have affected the magnitude of SRH service utilization reported. This study also did not ask about barriers that hinder HEWs from increasing youth service utilization. Additionally, we didn't perform a qualitative study to assess the effect of cultural barriers such as existing norms, beliefs, and gender related barriers on SRH service utilizing. However, using nationally representative data is one of the important strengths of the current study.

Conclusion

This study demonstrates that SRH service utilization of young girls in rural Ethiopia is very low. Our findings also indicate that HEWs have a significant contribution to improving SRH service utilization of young girls through health education during home visits, during school visits, and at HPs. However, more effort might be required to significantly increase the exposure of young girls to HEP services to increase service uptake. Encouraging young girls to build trust in HEWs and make young girls know what SRH related services are available at the HP is the first step toward improving service uptake. HEWs need to give special focus on younger girls and never married young girls to increase their service uptake.

Acknowledgment

We are very thankful to MERQ consultancy for granting us to access this data source for this study. We acknowledge Ms. Saron who thoroughly edited this manuscript for language usage, spelling and grammar. Pre-Publication Support Service (PREPSS) supported the development of this manuscript by providing pre-publication peer-review and copy editing.

Competing interests: The authors have declared that they do not have any competing interests exist.

For peer review only - http://bmjopen.bmj.com/site/about/guidelines.xhtml

Funding: The study was conducted by MERQ Consultancy PLC as part of a national assessment on the Health Extension Program of Ethiopia. The national assessment was financed through a grant from the Bill & Melinda Gates Foundation (INV010174). The funder provided financial support to MERQ that covered professional fees for leaders but did not have any additional role in the study design, data collection and analysis, decision to publish, or preparation of the manuscript. The specific roles of these authors are articulated in the "author contributions" section.

Author Contributions: AMT YK GM TLD contributed to the conceptualization, designing, and conduct of the national HEP assessment, MGJ TLD MJ YS conceptualized the idea coved within this paper, MJ drafted the MS, MB led the data analysis and was involved in the drafting of the MS. GM MGJ TLD supervised the write-up of the draft, all coauthors critically reviewed the drat MS and contributed intellectual inputs to improve the content of the MS and endorsed the final version of the MS to be submitted for possible publication.

Research Ethics Approval: This study involves human participants and was approved by an Ethics Committee(s) or Institutional Board(s), called Ethiopian public health association (EPHI)-1613/820.

Data sharing: Data are available upon reasonable request

Data availability: questionnaire and STATA do file

References

1. UN. Youth and the 2030 agenda for sustainable development. 2019. Available from: https://www.un.org/development/desa/youth/wp-content/uploads/sites/21/2018/12/WorldYouthReport-2030Agenda.pdf

2. United Nations. World Population Monitoring Adolescent and Youth Department of Economic and Social Affairs Population Division [Internet]. United Nations. 2012. Available from: http://www.un.org/en/development/desa/population/publications/pdf/fertility/12_66976_adolescents_and_youth.pdf

For peer review only - http://bmjopen.bmj.com/site/about/guidelines.xhtml

3. FMOH. Adolescence And Youth Health(AYH) Case Team | FMOH [Internet]. 2016 [cited 2021 Mar 23]. Available from: http://www.moh.gov.et/index.php/am/AYH

4. United Nations. World population prospects 2019. Department of Economic and Social Affairs. World Population Prospects 2019. 2019.

5. UNFPA. The Case for Investing in Young People as part of a National Poverty Reduction Strategy [Internet]. 2005. Available from: http://www.unfpa.org/webdav/site/global/shared/documents/publications/2005/case_youngpeople_eng.pdf

6. WHO. World health statistics overview:monitoring health for the SDGs. 2019. Available from: https://apps.who.int/iris/handle/10665/324835.

7. Dehne KL, Riedner G. Sexually transmitted infections among adolescents: The need for adequate health services. Reprod Health Matters. 2001;9(17):170–83. doi: 10.1016/s0968-8080(01)90021-7. PMID: 11468834.

8. Central Statistical Agency [Ethiopia], ICF International. Ethiopia Demographic and Health Survey 2011. Ethiop Water Sect Dev Progr [Internet]. 2012;1–452. Available from: https://www.usaid.gov/sites/default/files/documents/1860/Ethiopia DHS 2016 KIR - Final 10-17-2016.pdf

9. Amare T, Yeneabat T, Amare Y. A Systematic Review and Meta-Analysis of Epidemiology of Risky Sexual Behaviors in College and University Students in Ethiopia , 2018. *J Environ Public Health*. 2019 Mar 20;2019:4852130. doi: 10.1155/2019/4852130. PMID: 3101584

10. Ethiopian Public Health Institute (EPHI) [Ethiopia] and ICF. Ethiopia Mini 396 Demographic and Health Survey 2019: Final Report. Rockville, Maryland, USA: EPHI and 397 ICF 2021. Available from: https://www.unicef.org/ethiopia/media/1721/file/The%202019%20Ethiopia%20Mini%20Demographic%20and%20Health%20Survey%20.pdf

11. Birhan Z, Tushune K, Jebena MG. Sexual and reproductive health services use,

For peer review only

For peer review only - http://bmjopen.bmj.com/site/about/guidelines.xhtml

perceptions, and barriers among young people in southwest Oromia, Ethiopia. *Ethiopian journal of health sciences*. 2018 Jan 10;28(1):37-48.

12. kidanu, kiros G. Health Sector Development Program Four(HSDP-IV) Available from http://tucghe.org/HSDP%20IV.pdf

13. FMOH. Ethiopia second Generation Health Extension Program. 2015. Available from: https://publications.jsi.com/JSIInternet/Inc/Common/_download_pub.cfm?id=16833&lid=3

14. Alebachew Abebe, Waddington & Catriona.. Improving health system efficiency: Ethiopia human resources for health reforms. 2015. Available from: https://apps.who.int/iris/handle/10665/187240.

15. Kemal, Mohammed. Federal Democratic Republic of Ethiopia Ministry of Health Adolescent Reproductive Health Extension Package. Available from: https://www.researchgate.net/publication/332410714_Federal_Democratic_Republic_of_Ethiopia_Ministry_of_Health_Adolescent_Reproductive_Health_Extension_Package

16. Federal democratic republic of Ethiopia Ministry of Health, Health sector transformation Plan (HSTP). 2015. Available from: https://www.globalfinancingfacility.org/sites/gff_new/files/Ethiopia-health-system-transformation-plan.pdf

17. Merq conseltancy PPLC. HEP [National Assessment of The Ethiopian Health Extension Program] REPORT - MERQ Consultancy. 2021 [cited 2021 Nov 18]. p. 1–43. Available from: https://merqconsultancy.org/p/22/hep-national-assessment-of-the-ethiopian-health-extension-program-report

18. Yitayal M., Et.al. Health extension program factors, frequency of household visits and being model households, improved utilization of basic health services in Ethiopia. *BMC Health Serv Res*. 2014;14:156. Available from: http://ovidsp.ovid.com/ovidweb.cgi?T=JS&PAGE=reference&D=emed12&NEWS=N&AN=24708661

For peer review only - http://bmjopen.bmj.com/site/about/guidelines.xhtml

19. Ethiopia Central Statistical Agency (CSA), Ethiopia Demographic and Health Survey 2016, Central Statistical Agency, Addis Ababa & ICF International, Calverton, MD, USA, 2016.Available from: https://dhsprogram.com/pubs/pdf/FR328/FR328.pdf

20. Amanuel AA, Seme AS. Reproductive Health Knowledge and Services Utilization among Rural Adolescents in Machakal district, Northwest Ethiopia. *Asian Journal of Pharmacy*, Nursing and Medical Sciences. 2013 May 25;1(1).

21. Binu W, Marama T, Gerbaba M, Sinaga M. Sexual and reproductive health services utilization and associated factors among secondary school students in Nekemte town, Ethiopia. *Reprod Health*. 2018;Dec15(1):1–0. 22.

22. Gebreyesus H, Teweldemedhin M, Mamo A. Determinants of reproductive health services utilization among rural female adolescents in Asgede-Tsimbla district Northern Ethiopia: A community based cross-sectional study. *Reprod Health*. 2019;Dec16(1):1–10.

23. Ayehu A, Kassaw T HG. Level of young people sexual and reproductive health service utilization and its associated factors among young people in Awabel District, Northwest Ethiopia. *PLoS One*. 2016;Mar 18; 11(3): e0151613

24. Yitayal M, Berhane Y, Worku A, et.al. Health extension program factors , frequency of household visits and being model households , improved utilization of basic health services in Ethiopia. *BMC Health services research* 2014 Dec;14(1):1-9. doi: 10.1186/1472-6963-14-156

25. Na M, Jennings L, Talegawkar SA, Ahmed S. Association between women's empowerment and infant and child feeding practices in sub-Saharan Africa: an analysis of Demographic and Health Surveys. *Public health nutrition*. 2015. Dec;18(17): 3155–65.

26. Nair M, Baltag V, Bose K, Boschi-Pinto C, Lambrechts T, Mathai M. Improving the Quality of Health Care Services for adolescents , globally : a standards-driven approach. *Journal of Adolescent Health*. 2015 Sep 1;57(3):288-98.

27. FIGO. International Federation of Gynecology and Obstetrics. Ethical Issues in Obstetrics and Gynecology. FIGO Committee for the Study of Ethical Aspects of Human

For peer review only - http://bmjopen.bmj.com/site/about/guidelines.xhtml

Reproduction and Women's Health. London. 2012. 1–148 p.

28. ACHE Code of Ethics | American College of Healthcare Executives [Internet]. [cited 2021 Mar 24]. Available from: https://www.ache.org/about-ache/our-story/our-commitments/ethics/ache-code-of-ethics

29. Adolescent and Youth-Friendly Services | The Challenge Initiative [Internet]. [cited 2021 Jul 3]. Available from: https://tciurbanhealth.org/courses/adolescent-youth-sexual-reproductive-health-services-supply/lessons/adolescent-and-youth-friendly-services-3/

30. Denno DM, Hoopes AJ, Chandra-Mouli V. Effective strategies to provide adolescent sexual and reproductive health services and to increase demand and community support. *J Adolesc Health*. 2015;56(1):S22–41. Available from: http://dx.doi.org/10.1016/j.jadohealth.2014.09.012

31. Feleke SA, Koye DN, Demssie AF, Mengesha ZB. Reproductive health service utilization and associated factors among adolescents (15-19 years old) in Gondar town, Northwest Ethiopia. *BMC Health services resarch*. 2013;Dec;13(1):1–0.

32. Abebe M, Awoke W. Utilization of Youth Reproductive Health Services and Associated Factors among High School Students in Bahir Dar, Amhara Regional State, Ethiopia. *Open Journal of Epidemiology*. 2014;Mar27(02):69–75.

33. Haile B, Shegaze M, Feleke T, Glagn M, et.al. Disparities in utilization of sexual and reproductive health services among high school adolescents from youth friendly service implemented and non-implemented areas of Southern Ethiopia. *Arch Public Heal*. 2020 Dec 1 [cited 2021 Mar 23];78(1):126. Available from: https://archpublichealth.biomedcentral.com/articles/10.1186/s13690-020-00508-w

34. Amsale C, Yemane B. Knowledge of sexually transmitted infections and barriers to seeking health services among high school adolescents in Addis Ababa, Ethiopia. *Journal of AIDS and Clinical Research*. 2012;3(5).35.

35. Tilahun M, Mengistie B, Egata G, Reda AA. Health workers ' attitudes toward sexual and reproductive health services for unmarried adolescents in Ethiopia. *Reproductive health*.

For peer review only - http://bmjopen.bmj.com/site/about/guidelines.xhtml

2012;Dec;9(1):1–7, doi: https://doi.org/10.1186/1742-4755-9-19.

For peer review only

For peer review only - http://bmjopen.bmj.com/site/about/guidelines.xhtml

STROBE Statement—Checklist of items that should be included in reports of ***cross-sectional studies***

	Item No	Recommendation	Page No
**Title and abstract**	1	(*a*) Indicate the study's design with a commonly used term in the title or the abstract	1 - 2
		(*b*) Provide in the abstract an informative and balanced summary of what was done and what was found	1 - 2
**Introduction**			
Background/rationale	2	Explain the scientific background and rationale for the investigation being reported	3
Objectives	3	State specific objectives, including any prespecified hypotheses	4
**Methods**			
Study design	4	Present key elements of study design early in the paper	5
Setting	5	Describe the setting, locations, and relevant dates, including periods of recruitment, exposure, follow-up, and data collection	4-5
Participants	6	(*a*) Give the eligibility criteria, and the sources and methods of selection of participants	5
Variables	7	Clearly define all outcomes, exposures, predictors, potential confounders, and effect modifiers. Give diagnostic criteria, if applicable	6
Data sources/ measurement	8*	For each variable of interest, give sources of data and details of methods of assessment (measurement). Describe comparability of assessment methods if there is more than one group	5
Bias	9	Describe any efforts to address potential sources of bias	5-6
Study size	10	Explain how the study size was arrived at	5
Quantitative variables	11	Explain how quantitative variables were handled in the analyses. If applicable, describe which groupings were chosen and why	7
Statistical methods	12	(*a*) Describe all statistical methods, including those used to control for confounding	7
		(*b*) Describe any methods used to examine subgroups and interactions	7
		(*c*) Explain how missing data were addressed	7
		(*d*) If applicable, describe analytical methods taking account of sampling strategy	7
		(*e*) Describe any sensitivity analyses	7
**Results**			
Participants	13*	(a) Report numbers of individuals at each stage of study—eg numbers potentially eligible, examined for eligibility, confirmed eligible, included in the study, completing follow-up, and analysed	8
		(b) Give reasons for non-participation at each stage	8
		(c) Consider use of a flow diagram	8
Descriptive data	14*	(a) Give characteristics of study participants (eg demographic, clinical, social) and information on exposures and potential confounders	8
		(b) Indicate number of participants with missing data for each variable of interest	8
Outcome data	15*	Report numbers of outcome events or summary measures	10
Main results	16	(*a*) Give unadjusted estimates and, if applicable, confounder-adjusted estimates and their precision (eg, 95% confidence interval). Make clear which confounders were adjusted for and why they were included	10

For peer review only - http://bmjopen.bmj.com/site/about/guidelines.xhtml

		(*b*) Report category boundaries when continuous variables were categorized	10
		(*c*) If relevant, consider translating estimates of relative risk into absolute risk for a meaningful time period	11
Other analyses	17	Report other analyses done—eg analyses of subgroups and interactions, and sensitivity analyses	11
**Discussion**			
Key results	18	Summarise key results with reference to study objectives	11
Limitations	19	Discuss limitations of the study, taking into account sources of potential bias or imprecision. Discuss both direction and magnitude of any potential bias	13
Interpretation	20	Give a cautious overall interpretation of results considering objectives, limitations, multiplicity of analyses, results from similar studies, and other relevant evidence	11-13
Generalisability	21	Discuss the generalisability (external validity) of the study results	14
**Other information**			
Funding	22	Give the source of funding and the role of the funders for the present study and, if applicable, for the original study on which the present article is based	

*Give information separately for exposed and unexposed groups.

Note: An Explanation and Elaboration article discusses each checklist item and gives methodological background and published examples of transparent reporting. The STROBE checklist is best used in conjunction with this article (freely available on the Web sites of PLoS Medicine at http://www.plosmedicine.org/, Annals of Internal Medicine at http://www.annals.org/, and Epidemiology at http://www.epidem.com/). Information on the STROBE Initiative is available at www.strobe-statement.org.

For peer review only

For peer review only - http://bmjopen.bmj.com/site/about/guidelines.xhtml
